# Chiral information harvesting in helical poly(acetylene) derivatives using oligo(*p*-phenyleneethynylene)s as spacers†

**DOI:** 10.1039/d0sc02685a

**Published:** 2020-06-23

**Authors:** Zulema Fernández, Berta Fernández, Emilio Quiñoá, Ricardo Riguera, Félix Freire

**Affiliations:** Centro Singular de investigación en Química Biolóxica e Materiais Moleculares (CiQUS), Departamento de Química Orgánica, Universidade de Santiago de Compostela E-15782 Santiago de Compostela Spain felix.freire@usc.es; Departamento de Química Física, Universidade de Santiago de Compostela E-15782 Santiago de Compostela Spain

## Abstract

A chiral harvesting transmission mechanism is described in poly(acetylene)s bearing oligo(*p*-phenyleneethynylene)s (OPEs) used as rigid achiral spacers and derivatized with chiral pendant groups. The chiral moieties induce a positive or negative tilting degree in the stacking of OPE units along the polymer structure, which is further harvested by the polyene backbone adopting either a *P* or *M* helix.

## Abstract 2

A chiral harvesting transmission mechanism is described in poly(acetylene)s bearing oligo(*p*-phenyleneethynylene)s (OPEs) used as rigid achiral spacers and derivatized with chiral pendant groups.

## Graphical abstract

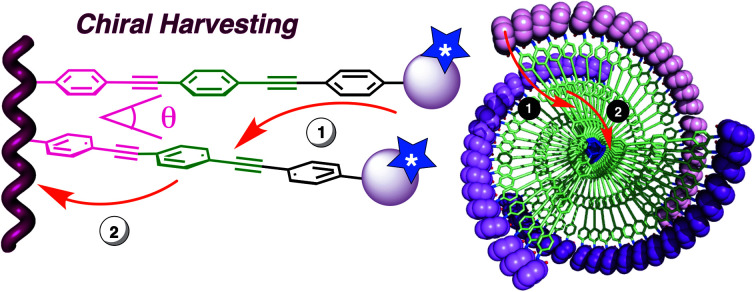

During the last years, dynamic helical polymers have attracted the attention of the scientific community due to the possibility of tuning the helical sense and/or the elongation of the helical structure by using external stimuli.^1–14^

In the case of a chiral dynamic helical polymer, modifications in its structure—helical sense enhancement or helix inversion—arise from conformational changes induced at its chiral pendants—usually, with just one stereocenter—, by stimuli such as variations in solvent polarity or temperature, the addition of certain ions, and so on (Fig. 1a).^15^ On the other hand, if a helical polymer is achiral (*i.e.*, bearing achiral pendants), the chiral amplification phenomena can emerge from interactions between the polymer and external chiral molecules.^16^ In both the above cases, the changes produced in the helical structures are related to the spatial dispositions adopted by the substituents or associated species at the pendant groups.^17–19^

**Fig. 1:**
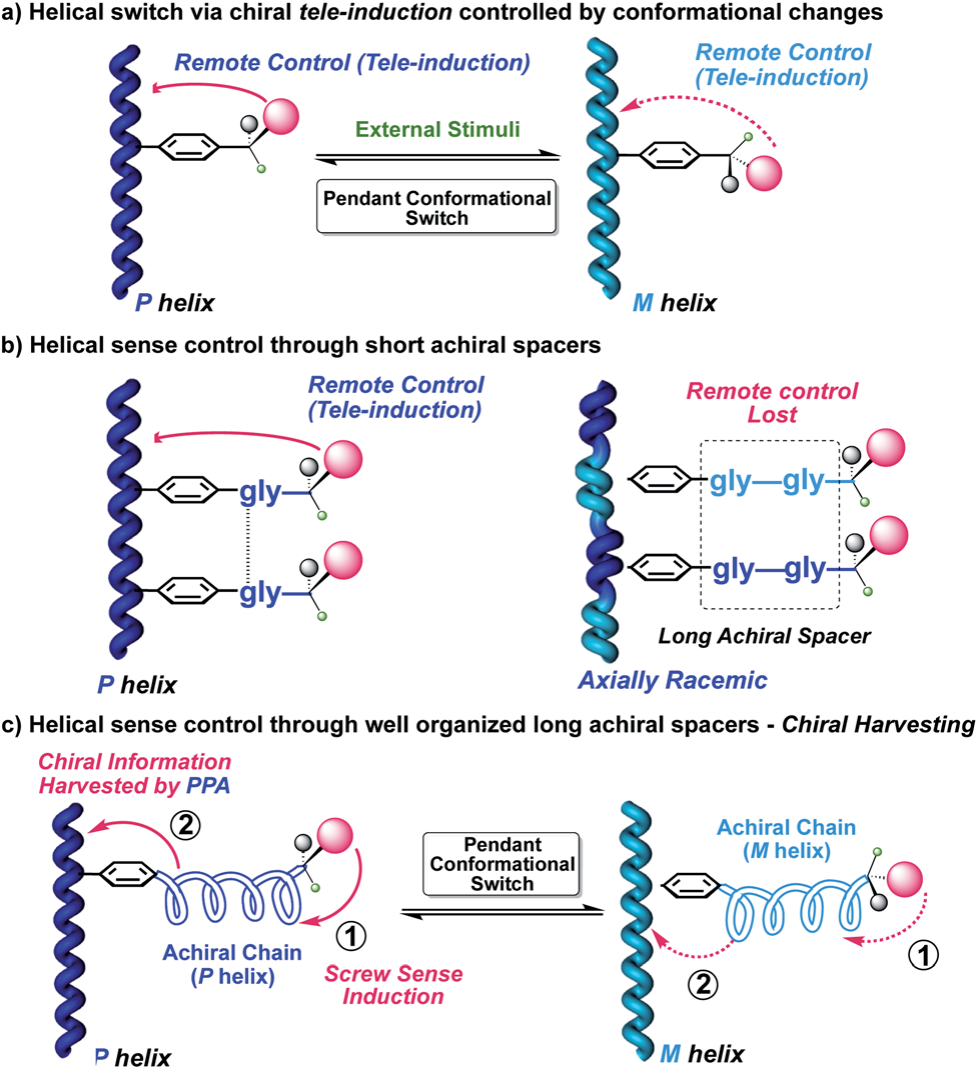
Several scenarios depicting conceptual representations of the transmission of chiral information. (a) Helical switch *via* chiral tele-induction. (b) Effect of distance on chiral tele-induction from multichiral pendants. (c) Helicity controlled by the conformational composition of achiral spacers.

A step forward in the helical sense control of poly(phenylacetylene)s (PPA)s is to study different mechanisms of transmission of chiral information from the pendant to the polyene backbone by introducing achiral spacers. The goal is to demonstrate how far it is possible to place the chiral center and still have an effective chiral induction on the polyene backbone. Therefore, transmission of the chiral information from a remote position can occur through space, thus overpassing the distance generated by the spacer—tele-induction—(Fig. 1b),^20–28^ or through the achiral spacer itself, producing in it a preferred structure, such as a helical structure and where the orientation of the achiral helix is further transmitted to the polyene backbone—conformational switch—(Fig. 1c).^29–31^

For the first mechanism—chiral tele-induction—, both flexible and rigid spacers have been designed.^20–28^ In all cases, supramolecular interactions, such as H bonding or π–π stacking, generate organized structures. As a result, the chiral center is located into a specific orientation, producing an effective helical induction. Additionally, those studies allow evaluating how distances and sizes have an effect on this phenomenon.

In the second strategy, the helix induction is transmitted through conformational changes along an achiral spacer which is harvested by the polyene. For instance, an achiral peptide or an achiral polymeric helix derivatized at one end with a chiral residue and linked to the polymer main chain at the other end. In such cases, changes in the absolute configuration or even just a conformational change at the chiral center can induce an opposite helical structure into the achiral spacer, which in turn will be harvested by the polymer main chain (Fig. 1c).^29–31^

Herein we will demonstrate another remote chiral induction mechanism based on a different chiral harvesting process. In this case, the chiral center does not produce a conformational change at the achiral spacer, but affects its array within the helical scaffold. Thus, to perform these studies we decided to introduce the use of oligo(*p*-phenyleneethynylene)s (*m* = 1, 2, 3) (OPEs) as rigid spacers to separate the distant chiral center from the polyene backbone. These OPE units have been used in the formation of benzene-1,3,5-tricarboxamide (BTA) based supramolecular helical polymers, demonstrating their ability to stack with a certain tilting degree commanded by the chiral center.^32–34^

Hence, in our design, the chiral moiety will determine the supramolecular chiral orientation of the OPE groups used as spacers, which is further harvested by the polyene backbone. The overall process yields a helix with a preferred screw sense (Fig. 2).

**Fig. 2:**
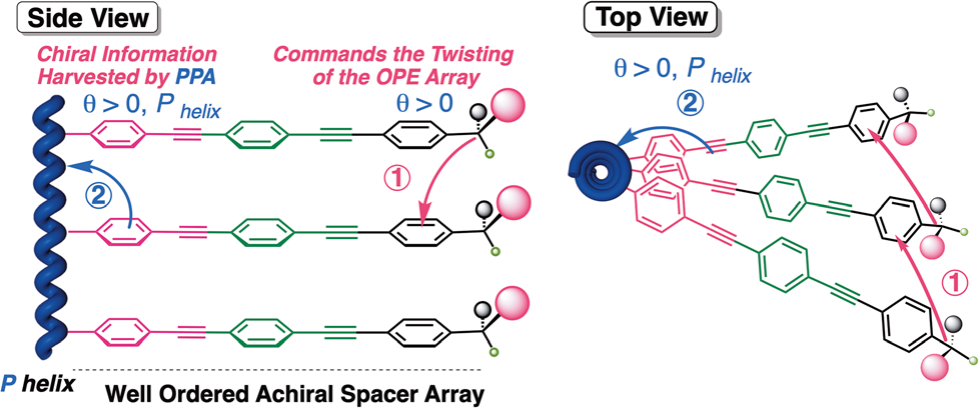
Conceptual side view and top view of the chiral information transmission mechanism from stereocenters at the far end of oligo(*p*-phenyleneethynylene) spacers to the polyene backbone *via* chiral harvesting.

To perform these studies, we used as model compounds two PPAs—poly-(*R*)-**1** and poly-(*S*)-**1**—derived from the 4-ethynylanilide of (*S*)- and (*R*)-α-methoxy-α-phenylacetic acid (MPA, m-(*S*/*R*)-**1**), whose helical structures and dynamic behaviors have been deeply studied by our group—poly-(*R*)-**1** and poly-(*S*)-**1**—(Fig. 3).^35–46^ By using these polymers as reference materials, four novel PPAs were designed introducing two OPE spacers—4-[(*p*-phenyleneethynylene)_*n*_]ethynylanilide (*n* = 1, 2)—between the phenyl acetylene group and the (*S*)- or (*R*)-α-methoxy-α-phenylacetic acid (MPA) chiral group. Thus, monomers m-(*S*)- and m-(*R*)-**2** and m-(*S*)- and m-(*R*)-**3** (Fig. 3a) were prepared and submitted to polymerization by using a Rh(i) catalyst poly-(*S*)- and poly-(*R*)-**2** and poly-(*S*)- and poly-(*R*)-**3** (Fig. 3b) were obtained in high yield and showed Raman spectra characteristic of *cis* polyene backbones (see Fig. S11 and S12†).

**Fig. 3:**
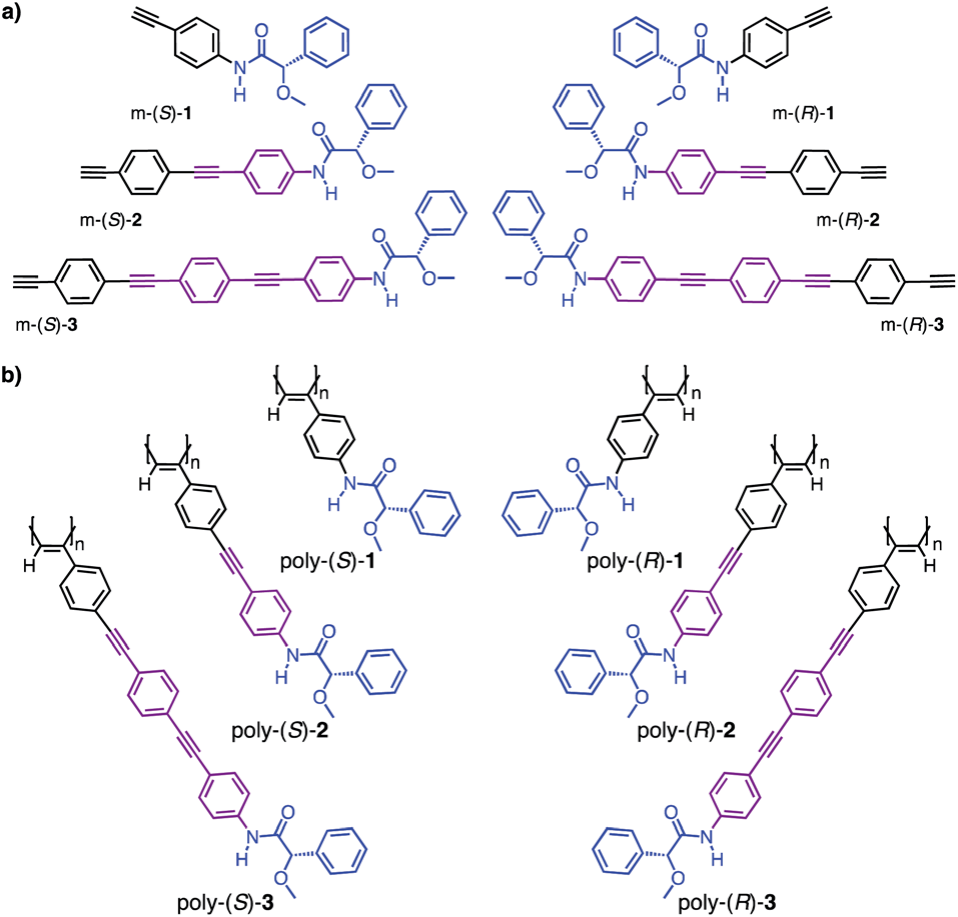
(a) Monomers and (b) polymers synthetized in this study.

X-ray structures of the monomers show a preferred *antiperiplanar* (*ap*) orientation between the carbonyl and methoxy groups (O=C–C–OMe) for m-(*R*)-**2** and m-(*S*)-**3**, whereas in the case of m-(*S*)-**1** a *synperiplanar* (*sp*) geometry is favoured (Fig. 4a).^35^ In complementary studies, CD spectra of monomers m-(*S*)-[**1–3**] in CHCl_3_ show negative Cotton effects, indicative of major *ap* conformations in solution (Fig. 4b),^35^ further corroborated by theoretical calculations (see Fig. S10†). Interestingly, the maximums of the Cotton effects in CD undergo a bathochromic shift—from 266 nm in m-**1** to 327 nm in m-**3**—due to a larger conjugation of the π electrons (from the anilide to the alkyne group) when the length of the spacer increases (Fig. 4b).

**Fig. 4:**
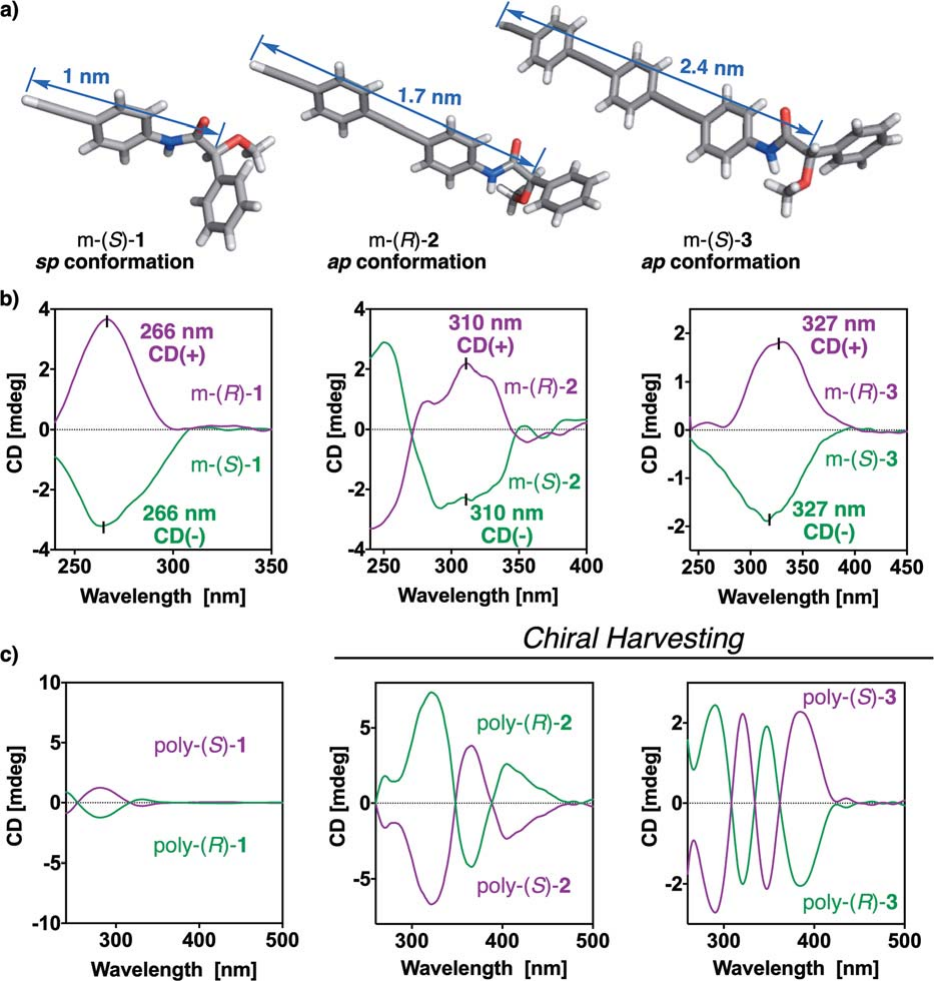
(a) X-ray structures of m-(*S*)-**1**, m-(*R*)-**2** and m-(*S*)-**3**. (b) CD traces of m-(*S*)- and m-(*R*)-**1**; m-(*S*)- and m-(*R*)-**2**; m-(*S*)- and m-(*R*)-**3** in CHCl_3_ (0.1 mg mL^−1^). (c) CD spectra for poly-(*S*)- and poly-(*R*)-**1** in CHCl_3_ (0.1 mg mL^−1^); poly-(*S*)- and poly-(*R*)-**2** in DMSO (0.1 mg mL^−1^); poly-(*S*)- and poly-(*R*)-**3** in DMSO (0.1 mg mL^−1^).

CD studies of the polymer series bearing OPE spacers—poly-(*R*)- and poly-(*S*)-[**2–3**]—in different solvents show the formation of a PPA helical structure with a preferred helical sense, while the parent polymer, poly-**1**, devoid of the OPE unit, has a poor CD. This is a very interesting phenomena that indicates that the OPE spacers work as transmitters of the chiral information from remote chiral centers to the polyene backbone—placed at 1.7 nm for poly-**2** and at 2.4 nm for poly-**3**—(Fig. 4a). These large distances between the chiral center and the polymer main chain mean that other mechanisms of chiral induction, such as chiral tele-induction effect, should be almost null in these cases.

In these two polymers (poly-**2** and poly-**3**), the chiral information transmission mechanism must occur in different sequential steps. First, the chiral centers possessing a major (*ap*) conformation induce a certain tilting degree (*θ*) in the achiral spacer array. This step resembles the helical induction mechanism found in supramolecular helical polymers bearing OPE units.^32–34^ Next, the chiral array induced in the OPE units is harvested by the polyene backbone, resulting in an effective *P* or *M* helix induction (Fig. 2).^34,47^

Additional structural studies were carried out in poly-(*S*)-**2** and poly-(*S*)-**3** to obtain an approximated secondary structure of these polymers and determine their dynamic behaviour.

From literature it is known that the conformational equilibrium of poly-**1** can be altered in solution by the presence of metal ions. The addition of monovalent ions (*e.g.*, Li^+^) stabilizes the *ap* conformer at the pendant group by cation–π interactions, while divalent ions (*e.g.*, Ca^2+^) stabilize the *sp* conformations by chelation with the methoxy and carbonyl groups.^36,38,39,43^ As a result, both the *P* or *M* helical senses can be selectively induced in poly-**1** by the action of metal ions.

Therefore, we decided to add different perchlorates of monovalent and divalent metal ions to solutions of poly-(*S*)-**2** and poly-(*S*)-**3** with the aim of determining the conformational composition at the pendant groups. Thus, when monovalent metal ions (Li^+^, Ag^+^ and Na^+^) are added to a chloroform solution of poly-(*S*)-**2**, a chiral enhancement is observed (Fig. 5d for Li^+^ and Fig. S16† for Na^+^ and Ag^+^). IR and ^7^Li-NMR studies show that those ions stabilize the *ap* conformer at the pendant group in a similar fashion to poly-**1**, this is by coordination to the carbonyl group of the MPA (Fig. 5g) and the presence of a cation–π interaction with the aryl ring of the chiral (|Δ*δ*| ^7^Li *ca.*, 3.75 ppm) (Fig. 5f and ESI†). Therefore, addition of Li^+^ produces a larger number of pendant groups with *ap* conformation among poly-**2,** which triggers a chiral enhancement effect through a cooperative process.

**Fig. 5:**
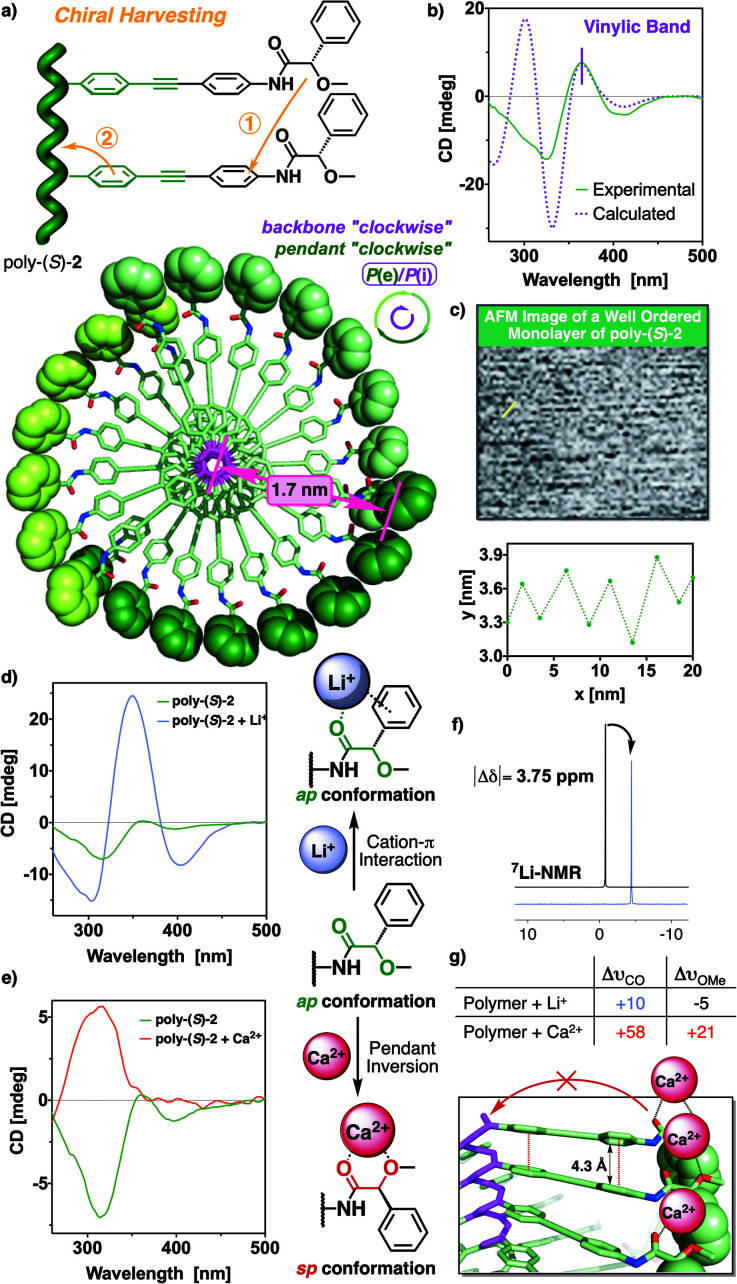
(a) Conceptual representation of the chiral information harvesting and top view of the 3D model for poly-(*S*)-**2**. (b) CD spectra of poly-(*S*)-**2** (0.2 mg mL^−1^) in DMSO *vs.* calculated ECD spectra. Full width at half-maximum (FWHM) equals 20 nm. (c) Low-resolution AFM image from a poly-(*S*)-**2** monolayer and profile depicting the chain separation of the yellow highlighted area in the AFM image. (d) CD spectra showing the chiral enhancement after the addition of Li^+^ (50 mg mL^−1^, THF) to a poly-(*S*)-**2** solution (0.1 mg mL^−1^, THF). (e) CD trace of poly-(*S*)-**2** before and after the addition of a Ca^2+^ solution (50 mg mL^−1^, THF). (f) ^7^Li-NMR spectra substantiating the cation–π interaction. (g) IR shifts observed for carbonyl and methoxy groups after the addition of LiClO_4_ and Ca(ClO_4_)_2_ (50 mg mL^−1^, THF) to a poly-(*S*)-**2** solution (3 mg mL^−1^, CHCl_3_). The coordination modes of the MPA moiety with Li^+^ and Ca^2+^ are shown vertically in the middle of the figure.

On the contrary, the addition of perchlorates of divalent metal ions, such as Ca^2+^and Zn^2+^, produced an inversion of the third Cotton band—310 nm—associated to the MPA moiety and the disappearance of both first and second Cotton effects (Fig. 5e for Ca^2+^ and Fig. S17† for Zn^2+^). This is a very interesting outcome because, although the conformational equilibrium at the MPA group changes from *ap* to *sp* after the addition of Ca^2+^, the number of pendant groups with *sp* conformation do not reach the number needed to trigger the helix inversion process and in fact, a mixture of *P* and *M* helices at the polyene backbone is obtained.

The helical structures adopted by both polymer systems, PPAs (poly-**1**) and poly[oligo(*p*-phenyleneethynylene)phenylacetylene]s (POPEPAs) (poly-**2** and poly-**3**), are defined by two coaxial helices, one formed by the polyene backbone (internal helix, CD active) and the other constituted by the pendants (external helix, observed by AFM).

These two helices can rotate in either the same or the opposite sense, depending on the dihedral angle between conjugated double bonds. Thus, internal and external helices rotate in the same direction in *cis*-cisoidal polymers, while they rotate in opposite directions in *cis*-transoidal ones.^14,42,48,49^

In order to find out an approximated helical structure for poly-(*S*)-**2**, DSC studies were performed. The thermogram shows a compressed *cis*-cisoidal polyene skeleton (see Fig. S13a†), similar to the one obtained for poly-**1**.^42^ Moreover, AFM studies on a 2D crystal of poly-(*S*)-**2** did not produce high-resolution AFM images, although some parameters such as helical pitch (*c.a.*, 2.8 nm) and packing distance between helices of (*c.a.*, 6 nm) could be extracted from the well-ordered monolayer analyzed (Fig. 5c).

Previous structural studies in PPAs found that it is possible to correlate the internal helical sense with the Cotton band associated to the polyene backbone—CD (+), *P*_int_; CD (−), *M*_int_—.^50,51^ Herein, the positive Cotton effect observed for the polyene backbone [CD_365 nm_ = (+)] in poly-(*S*)-**2** is indicative of a *P* orientation of the internal helix, which correlates with a *P* orientation of the external helix in a *cis*-cisoidal polyene scaffold. To summarize, DSC, AFM and CD studies agree that poly-(*S*)-**2** is made up of a *cis*-cisoidal framework with *P*_int_ and *P*_ext_ helicities (Fig. 5a).

Computational studies [TD-DFT(CAM-B3LYP)/3-21G] were carried out on a *P* helix of an *n* = 9 oligomer of poly-(*S*)-**2**, possessing a *cis*-cisoidal polyene skeleton (*ω*_1_ = +50°, *ω*_3_ = −40°) and an *antiperiplanar* orientation of the carbonyl and methoxy groups at the pendants. The theoretical ECD spectrum obtained from these studies (Fig. 5b and see ESI† for additional information) is in good agreement with the experimental one, indicating that our model structure is a good approximation of the helical structure adopted by poly-(*S*)-**2**.

Next, a similar set of DSC and AFM studies were carried out for poly-(*S*)-**3**, that bears an OPE spacer with *n* = 2. The data showed that this polymer presents a compressed *cis*-cisoidal polyene skeleton, similar to those obtained for poly-**1** and poly-**2** (see Fig. S13b†), with a helical pitch of 3.8 nm and a *P*_ext_ helical sense (Fig. 6a and c).

**Fig. 6:**
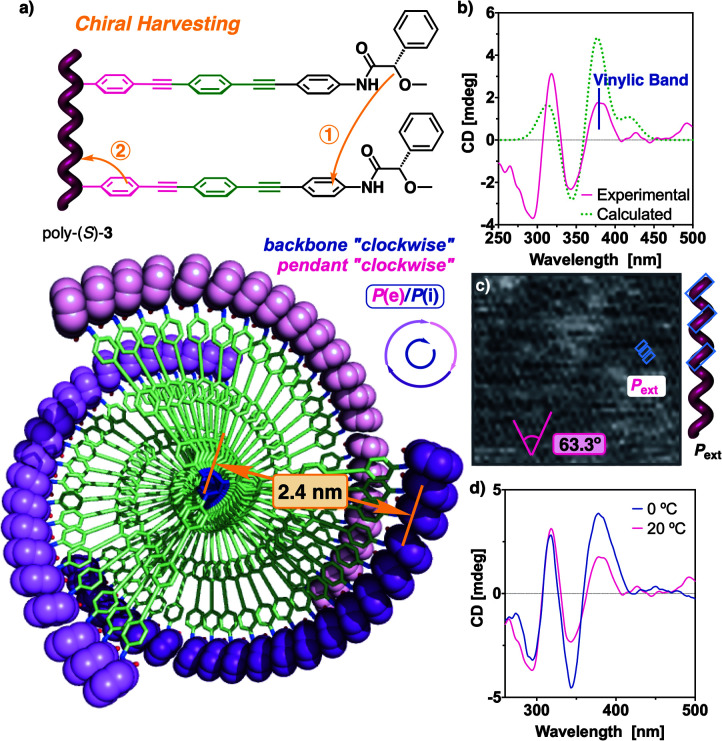
(a) Conceptual representation of the chiral information harvesting and top view of the 3D model for poly-(*S*)-**3**. (b) CD spectrum of poly-(*S*)-**3** in THF (0.2 mg mL^−1^) and comparison to the calculated ECD spectra. Full width at half-maximum (FWHM) equals 20 nm. (c) AFM image obtained from a poly-(*S*)-**3** monolayer. (d) CD traces for poly-(*S*)-**3** in THF polymerized at different temperatures.

UV studies indicate that, in poly-(*S*)-**3**, the polyene backbone absorbs at *ca.* 380 nm, coincident with the first Cotton effect, that is positive (see Fig. S15b†). Therefore, it reveals that poly-(*S*)-**3** adopts a *P*_int_ helicity (Fig. 6b). Thus, as expected for *cis*-cisoidal scaffolds, the orientations of the two coaxial helices are coincident.

Computational studies [TD-DFT(CAM-B3LYP)/3-21G] were carried out on a *P* helix of an *n* = 9 oligomer of poly-(*S*)-**3**, possessing a *cis*-cisoidal polyene skeleton (*ω*_1_ = +63°, *ω*_3_ = −40°) and an *antiperiplanar* orientation of the carbonyl and methoxy groups at the pendants. The theoretical results (Fig. 6b and see ESI† for additional information) match with the experimental data, indicating that our model structure is a good approximation to the helical structure adopted by poly-(*S*)-**3**.

Finally, the stimuli response properties of poly-(*S*)-**3** were explored by CD. These experiments revealed that the addition of monovalent or divalent metal ions to a chloroform solution of poly-(*S*)-**3** does not produce any significant effect in the structural equilibrium of this polymer (see Fig. S18†). This fact, in addition to the previous results obtained from the interaction of poly-(*S*)-**2** with divalent metal ions, corroborates the decrease of the dynamic character of helical PPAs when large OPEs are used as spacers.

The poor dynamic behaviour was further demonstrated by polymerizing m-(*S*)-**3** at a lower temperature (0 °C) (Fig. 6d). In this case, the region around 240–350 nm remains unaffected, indicating that the pendant is ordered in a similar manner in both batches of polymers, regardless of the temperature at which they were synthesized (20 °C and 0 °C). Interestingly, the magnitude of the first Cotton band is duplicated when the polymer is obtained at low temperature due to a stronger helical sense induction at the polyene backbone. This result indicates that a preorganization process may occur during polymerization, affecting the screw sense excess of the PPA.

In conclusion, a novel chiral harvesting transmission mechanism has been described in poly(acetylene)s bearing oligo(*p*-phenylenethynylene)s as rigid spacers that place the chiral pendant group away from the polyene backbone, at a distance around *ca.* 1.7 nm for poly-**2**, and 2.4 nm for poly-**3**. Hence, the disposition of the chiral moiety affects the stacking of the OPE units within the helical structure, inducing a specific positive or negative tilting degree, which is further harvested by the polyene backbone inducing either a *P* or *M* internal helix.

We believe that these results open new horizons in the development of novel helical structures by combining information from the helical polymers and supramolecular helical polymers fields, which leads to the formation of novel materials with applications in important fields such as asymmetric synthesis, chiral recognition or chiral stationary phases among others.

## Conflicts of interest

There are no conflicts to declare.

## Supplementary Material

SC-011-D0SC02685A-s001

SC-011-D0SC02685A-s002
