# Supplementary material for: Chiral information harvesting in helical poly(acetylene) derivatives using oligo(p-phenyleneethynylene)s as spacers
Source: Chem Sci. 2020 Jun 23;11(27):7182–7. doi: 10.1039/d0sc02685a (PMC8159296; doi:10.1039/d0sc02685a)
Supplement: SC-011-D0SC02685A-s001 [file SC-011-D0SC02685A-s001.pdf]

Electronic Supplementary Material (ESI) for Chemical Science.
This journal is © The Royal Society of Chemistry 2020

Chiral Information Harvesting in Poly(acetylene)s Derivatives Using Oligo(*p*-phenyleneethynylene)s as Spacers

Zulema Fernández, Berta Fernández, Emilio Quiñoá, Ricardo Riguera and Félix Freire*

Center for Research in Biological Chemistry and Molecular Materials (CiQUS) and Department of Organic Chemistry. University of Santiago de Compostela, 15782, Santiago de Compostela, Spain.

felix.freire@usc.es

http://felixfreire.com

Supporting Information

Contents

1. MATERIALS AND METHODS 2

2. SYNTHESIS OF THE MONOMERS 4

3. CRYSTALLOGRAPHIC DATA 15

4. THEORETICAL CALCULATIONS FOR MONOMERS 18

5. SYNTHESIS OF POLYMERS 18

5.1 General Procedure for Polymerization 18

5.2 Low Temperature Polymerization 19

5.3 NMR and Raman Experiments 19

6. GPC STUDIES 20

7. THERMAL STUDIES 20

7.1 DSC Studies 20

7.2 TGA Studies 20

8. CONFORMATIONAL STUDIES 21

8.1 Behavior in Solution 21

8.2 Response to External Stimuli: Interaction with Metals 21

9. ATR/FT-IR STUDIES 25

10. CATION-Π EXPERIMENTS 26

12. LOW TEMPERATURE POLYMERIZATION 26

11. VT-CD EXPERIMENTS 27

12. ATOMIC FORCE MICROSCOPY (AFM) MEASUREMENTS **28**

13. THEORETICAL CALCULATIONS FOR POLYMERS **30**

13.1 Computational Details **30**

13.2 Additional Computational Details **33**

14. SUPPORTING REFERENCES **34**

1. Materials and Methods

CD measurements were done in a Jasco-720 and UV spectra were registered in a Jasco V-630. The amount of polymer used is indicated in the corresponding section. Measurements were performed in a 1mm quartz cell. The concentration of perchlorate salts used in the studies was 50 mg mL^{-1} in THF. The concentration of the ionic salts used in the studies was 100 mg mL^{-1} in dry THF.

VT-CD measurements were performed in a Jasco-1100.

IR spectra were recorded in a Perkin Elmer FT-IR ATR Spectrum Two.

The optical rotation was measured in a Jasco P-2000.

Raman spectra were carried out in a Renishaw confocal Raman spectrometer (Invia Reflex model) equipped with a 785 nm diode laser and a 514 nm Ar laser.

DSC traces were obtained in a DSC Q200 Tzero Technology (TA Instruments, New Castle, UK) equipped with a refrigerated cooling system RCS90 (TA Instruments, New Castle, UK), using a Tzero low-mass aluminium pan.

TGA traces were obtained in a TGA Q5000 (TA Instruments, New Castle, UK) using a platinum pan.

Chiral HPLC experiments were carried out in a Waters System equipped with a Phenomenex Lux 5μm i-Amilose-1 column. The amount of monomer used was 0.5 mg mL^{-1} and the mixture hexane:isopropanol (8:2) was used as eluent (flow rate: 0.5 mL min^{-1}).

GPC studies were carried out in a Waters Alliance equipped with Phenomenex GPC columns (10^3 Å, 10^4 Å and 10^5 Å). The amount of polymer used was 0.5 mg mL^{-1}. THF was used as eluent (flow rate: 1 mL min^{-1}) and as inner standard, polystyrene narrow standards (PSS) were used.

AFM measurements were performed in a Multimode V Scanning Probe Microscope (Veeco Instruments) in air at r.t., with standard silicon cantilevers and supersharp cantilevers in tapping mode using 12 μm and 1 μm scanners. Nanoscope processing software and WSxM 4.0 Beta 1.0 [4] (Nanotec Electrónica, S.L.) was used for image analysis. All measurements were performed at CACTI (Vigo University, Spain).

The monolayers were prepared by the Langmuir-Schaefer method in a rectangular shallow trough made of poly(tetrafluoroethylene) (36 cm x 11 cm x 1 cm). A Wilhelmy balance provided with a 20 mm wide filter paper was used as a plate to measure the surface pressure. The polymer solution [250 µL (0.1 mg mL^{-1})] was spread drop by drop on the Milli-Q water surface. After fifteen minutes, time needed to allow the $CHCl_3$ evaporation as well as for the stabilization of the system, the barriers were gradually closed until reaching the target pressure (1 mN m^{-1}), indicative for the formation of a compact monolayer. This monolayer was recollected onto a freshly cleaved HOPG substrate (Telstar Instrumat, ZYH grade), with a dipper speed of $1 mm\cdot s^{-1}$.

Spartan 10 (MMFF94) was used for molecular modelling.

PyMOL was used as a molecular visualization system.

2. Synthesis of the Monomers

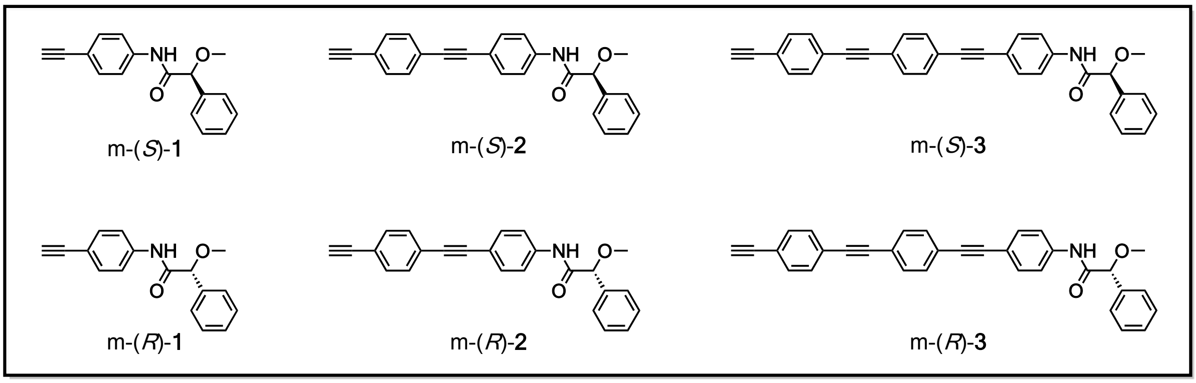

m-(*S*)-**1** and m-(*R*)-**1** were prepared according to the previously reported procedure.[S1]

Synthesis of 4-((4-((trimethylsilyl)ethynyl)phenyl)ethynyl)aniline (**1**)

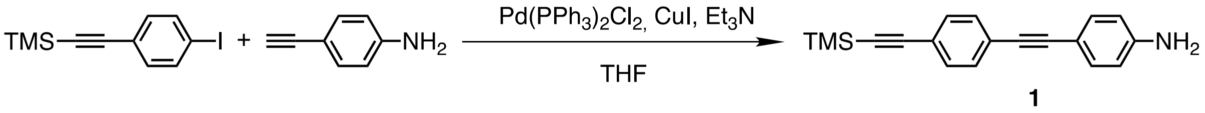

((4-iodophenyl)ethynyl)trimethylsilane (0.80 g, 2.66 mmol 1.00 equiv.), bis(triphenylphosphine)palladium(II) dichloride ($Pd(PPh_3)_2Cl_2$, 0.04 g, 0.05 mmol, 0.02 equiv.) and copper iodide (CuI, 0.01 g, 0.05 mmol, 0.02 equiv.) were dissolved in dry THF (30 mL). Next triethylamine (Et_3N, 15 mL) and 4-ethynylaniline (0.31 g, 2.66 mmol, 1 equiv.) were added and the mixture was stirred for three hours. After removing the solvent, the crude product was chromatographed on silica gel (70-230 mesh) with hexane/ethyl acetate (80:20) as eluent obtaining, after solvent removal, a pale-yellow solid (0.65 g, 84% of yield).

^{1}H NMR (300 MHz, $CDCl_3$) δ_H (ppm): 7.42 (s, 4H), 7.33 (d, 2H), 6.62 (d, 2H), 3.84 (s, 2H), 0.27 (s, 9H).

^{13}C NMR (75 MHz, $CDCl_3$) δ_C (ppm): 146.9, 133.0, 131.8, 131.1, 124.1, 122.1, 114.7, 112.2, 104.9, 95.9, 92.3, 87.1, 0.0.

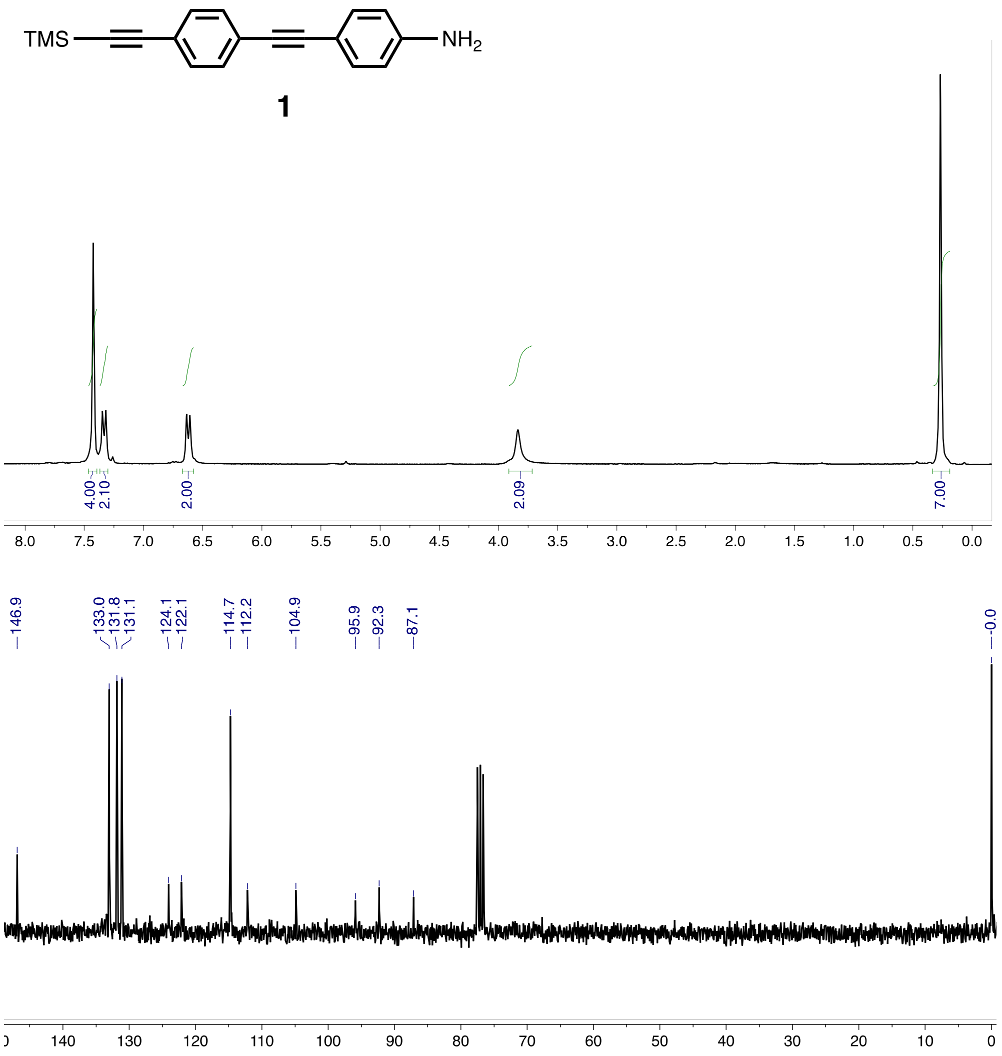

Figure S1. ^{1}H-NMR and ^{13}C-NMR of **1** in $CDCl_3$.

Synthesis of 4-((4-ethynylphenyl)ethynyl)aniline (**2**)

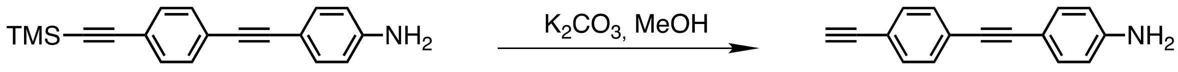

1 **2**

Once obtained the 4-((4-((trimethylsilyl)ethynyl)phenyl)ethynyl)aniline (**1**, 0.65 g, 2.24 mmol, 1.00 equiv.), it was dissolved in a mixture THF:MeOH (1:1) (10 mL) and potassium carbonate was added (K_2CO_3, 0.93 g, 931.10 mmol, 3.00 equiv.). After 15 minutes the organic layer was washed three times with water and dried over anhydrous $NaHCO_3$. The crude was chromatographed on silica gel (70-230 mesh) with hexane/ethyl acetate (70:30) as eluent (0.41 g, 70% of yield).

^{1}H NMR (300 MHz, $CHCl_3$) $\delta_{\square}$ (ppm): 7.44 (s, 4H), 7.33 (d, 2H), 6.63 (d, 2H), 3.85 (s, 2H), 3.16 (s, 1 H).

^{13}C NMR (75 MHz, $CHCl_3$) δ_C (ppm): 146.9, 133.0, 132.0, 131.2, 124.5, 121.1, 114.7, 112.2, 92.3, 86.9, 83.5, 78.6.

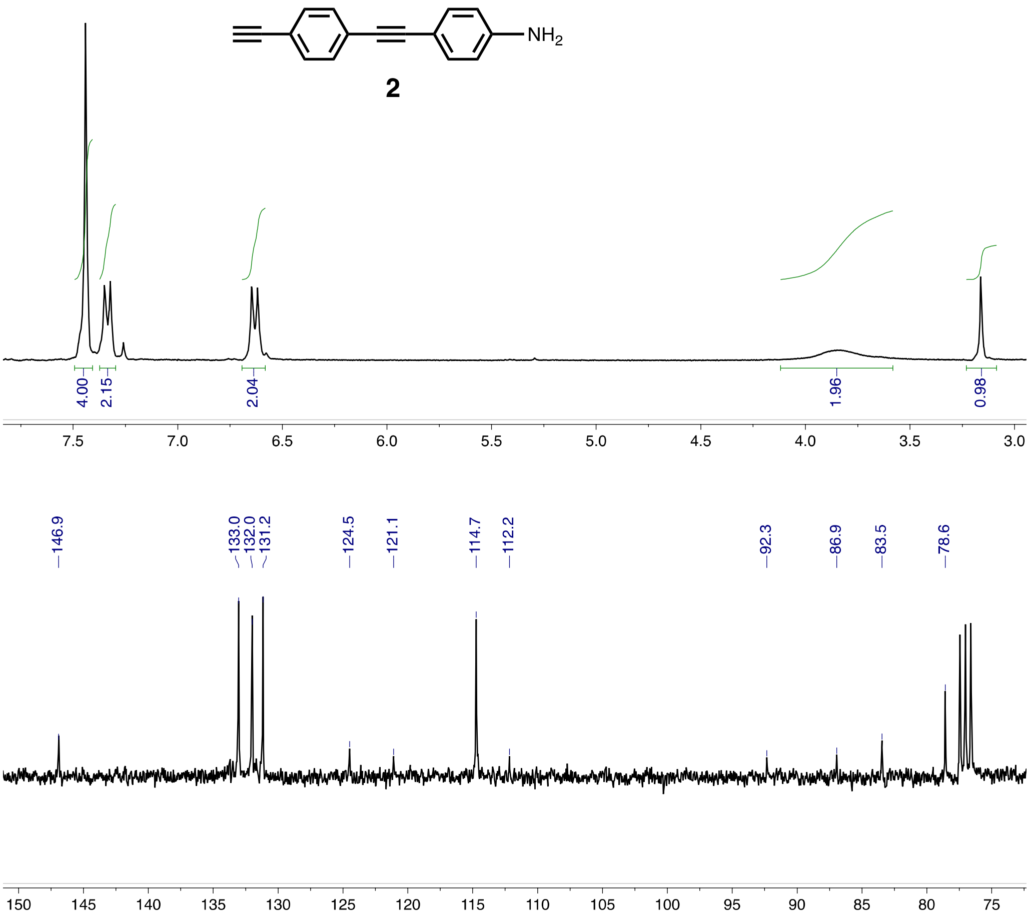

Figure S2. ^{1}H-NMR and ^{13}C-NMR of **2** in $CDCl_3$.

Synthesis of (*S*)- or (*R*)-*N*-(4-((4-ethynylphenyl)ethynyl)phenyl)-2-methoxy-2-phenylacetamide (m-(*S*)-**2** or m-(*R*)-**2**)

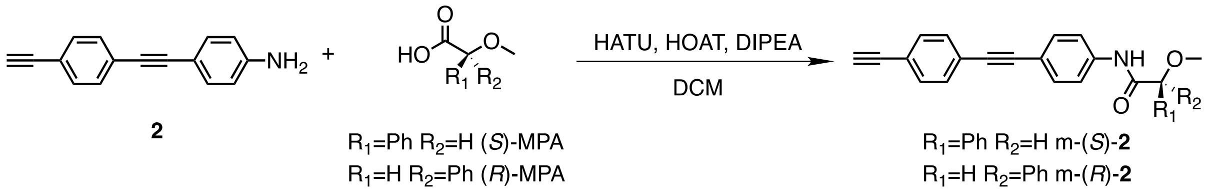

The corresponding (*S*) or (*R*)-α-methoxy-α-phenylacetic acid (0.28 g, 1.66 mmol, 1.20 equiv.), 2-(7-aza-1H-benzotriazole-1-yl)-1,1,3,3-tetramethyluronium (HATU, 0.63 g, 1.66 mmol, 1.20 equiv.), 1-hydroxy-7-azabenzotriazole (HOAt, 0.23 g, 1.66 mmol, 1.20 equiv.) and diisopropyltriethylamine (DIEA, 0.30 mL, 1.66 mmol, 1.20 equiv.) were dissolved in 20 mL of dry CH_2Cl_2. After 15 minutes, time needed to activate the acid, 4-((4-ethynylphenyl)ethynyl)aniline (0.30 g, 1.38 mmol, 1.00 equiv.) was added and the mixture was stirred overnight. The organic layer was washed three times with HCl 1M, a saturated solution of $NaHCO_3$ and brine. The combined organic layers were dried over anhydrous Na_2SO_4, filtered and evaporated at reduced pressure. The crude product was chromatographed on silica gel (70-230 mesh) with hexane/ethyl acetate (80:20) as eluent (0.60 g, 93% of yield).

^{1}H NMR (300 MHz, $CDCl_3$) δ_H (ppm): 8.65 (s, 1H), 7.62 (d, 2H), 7.55-7.43 (m, 8H), 7.39 (d, 3H), 4.75 (s, 1H), 3.45 (s, 3H), 3.18 (s, 1H).

^{13}C NMR (75 MHz, $CDCl_3$) δ_C (ppm): 168.6, 137.6, 136.4, 132.5, 132.1, 131.4, 128.7, 127.1, 123.8, 121.7, 119.4, 118.7, 91.2, 88.6, 83.3, 83.3, 78.9, 57.4.

HRMS (ESI) m/z calcd for $C_{25}H_{19}NO_2Na$ $[M+Na]^+$: 388.1308, found: 388.1308.

For m-(*S*)-**2**, $[a]_D^{20} =$ +5.59 (c = 5mg·mL^{-1}, DCM)

For m-(*R*)-**2**, $[a]_D^{20} =$ -4.96 (c = 5mg·mL^{-1}, DCM)

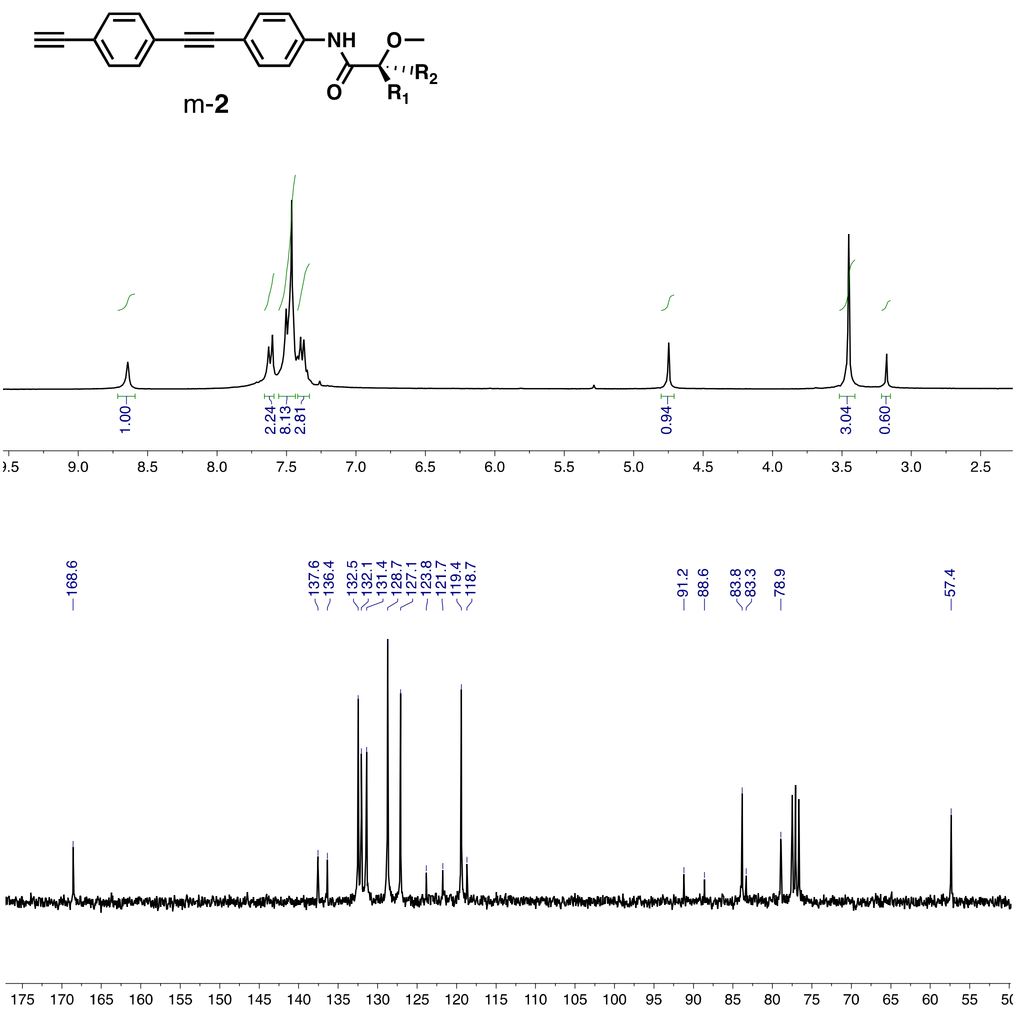

Figure S3. ^{1}H-NMR and ^{13}C-NMR of m-**2** in $CDCl_3$.

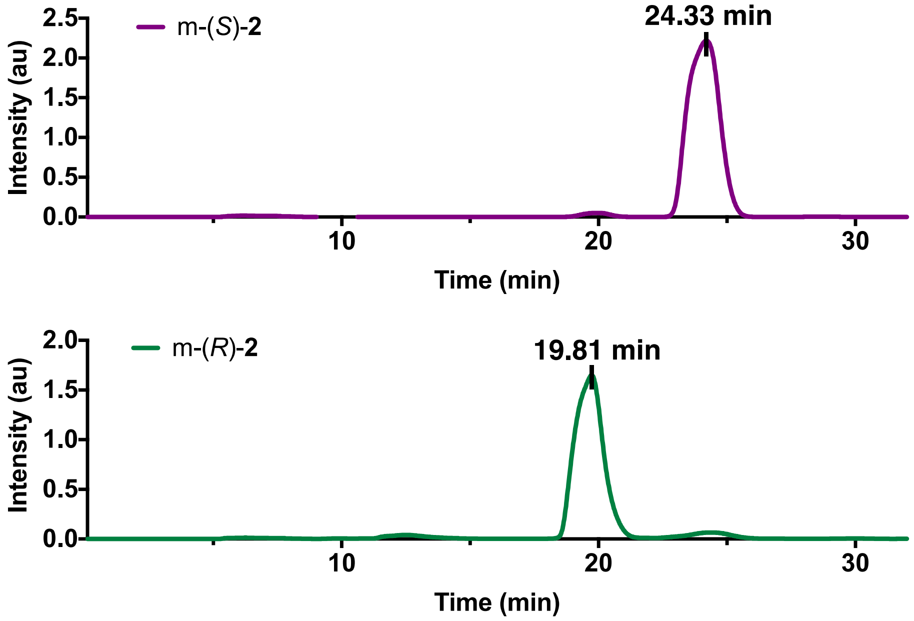

Figure S4. Chiral HPLC traces for m-(*S*)-**2** and m-(*R*)-**2** respectively.

Synthesis of (*S*)- or (*R*)-2-methoxy-2-phenyl-*N*-(4-((4-((4 ((trimethylsilyl)ethynyl)phenyl)ethynyl)phenyl)ethynyl)phenyl)acetamide ((*S*)-**3** or (*R*)-**3**)

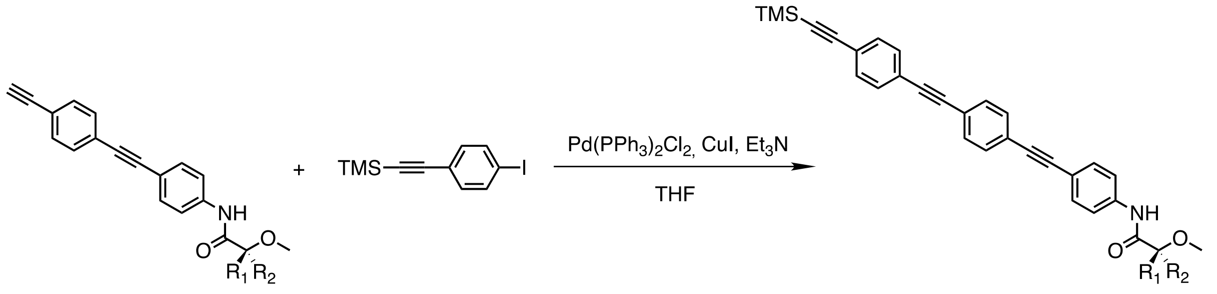

R_1=Ph R_2=H m-(*S*)-**2**
R_1=H R_2=Ph m-(*R*)-**2**

R_1=Ph R_2=H (*S*)-**3**
R_1=H R_2=Ph (*R*)-**3**

((4-iodophenyl)ethynyl)trimethylsilane (0.41 g, 1.36 mmol, 1.00 equiv.), bis(triphenylphosphine)palladium(II) dichloride ($Pd(PPh_3)_2Cl_2$, 0.02 g, 0.03 mmol, 0.02 equiv.) and copper iodide (CuI, 0.01 g, 0.03 mmol, 0.02 equiv.) were dissolved in dry THF (20 mL). Next triethylamine (Et_3N, 5 mL) and the corresponding (*S*)- or (*R*)-*N*-(4-((4-ethynylphenyl)ethynyl)phenyl)-2-methoxy-2-phenylacetamide (m-(*S*)-**2** or m-(*R*)-**2**, 0.50 g, 1.36 mmol, 1.00 equiv.) were added and the mixture was stirred at r.t. for three hours. After removing the solvent, the crude product was chromatographed on silica gel (70-230 mesh) with hexane/ethyl acetate (80:20) as eluent (0.42 g, 65% of yield).

^{1}H NMR (300 MHz, $CDCl_3$) δ_H (ppm): 8.64 (s, 1H), 7.62 (d, 2H), 7.54-7.44 (m, 12H), 7.40 (d, 3H), 4.75 (s, 1H), 3.45 (s, 3H), 0.27 (s, 9H).

^{13}C NMR (75 MHz, $CDCl_3$) δ_C (ppm): 168.5, 137.5, 136.4, 134.8, 132.4, 131.9, 131.5, 131.5, 131.4, 128.7, 127.1, 123.4, 123.1, 122.7, 119.4, 118.7, 104.6, 96.4, 91.3, 91.1, 90.9, 88.8, 83.8, 57.3, 0.0.

HRMS (ESI) m/z calcd for $C_{36}H_{32}NO_2Si$ $[M+H]^+$: 538.2124, found: 538.1308.

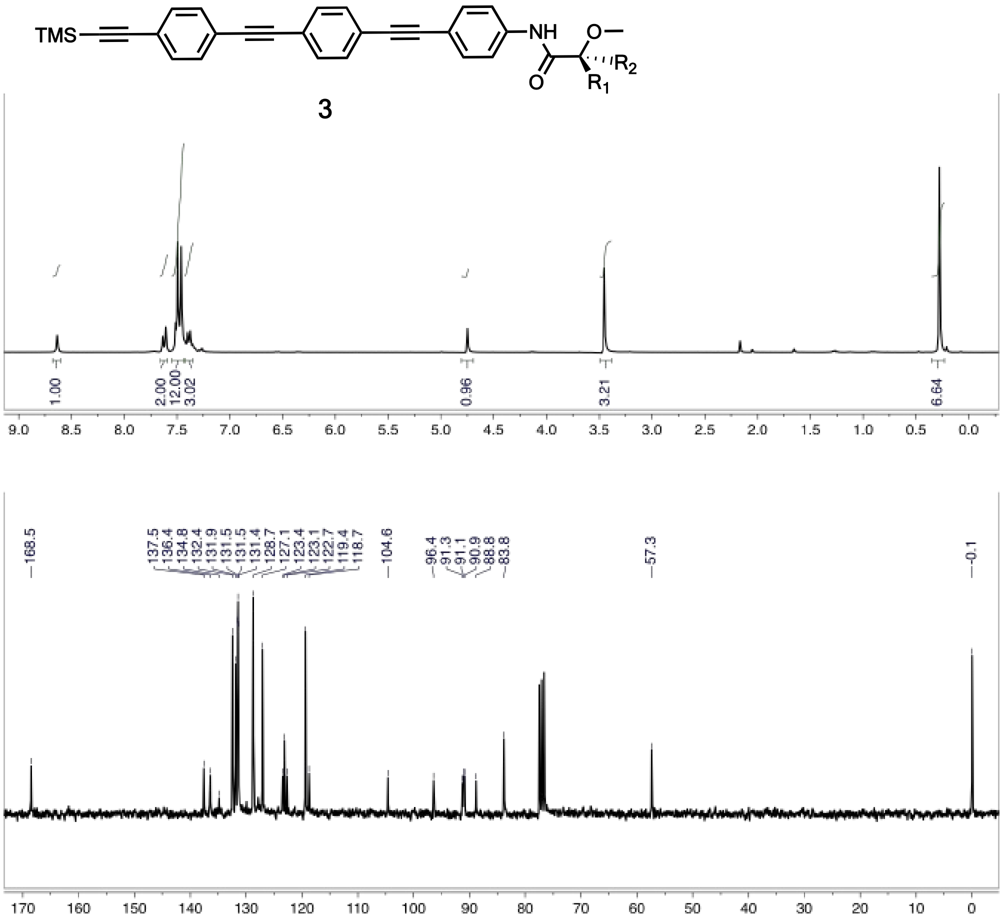

Figure S5. ^{1}H-NMR and ^{13}C-NMR of **3** in $CDCl_3$.

Synthesis of (*S*)- or (*R*)-*N*-(4-((4-((4-ethynylphenyl)ethynyl)phenyl)ethynyl)phenyl)-2-methoxy-2-phenylacetamide (m-(*S*)-**3** or m-(*R*)-**3**)

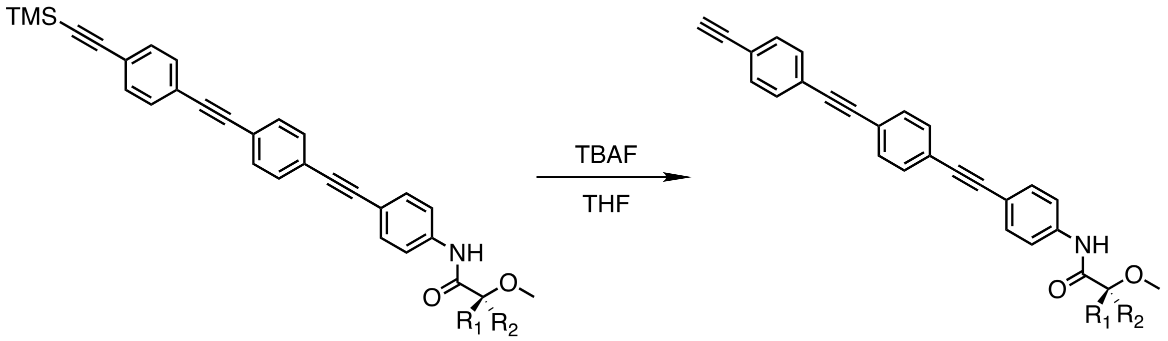

R_1=Ph R_2=H (*S*)-**3**
R_1=H R_2=Ph (*R*)-**3**

R_1=Ph R_2=H m-(*S*)-**3**
R_1=H R_2=Ph m-(*R*)-**3**

The corresponding (*S*)- or (*R*)-2-methoxy-2-phenyl-*N*-(4-((4-((4 ((trimethylsilyl)ethynyl)phenyl)ethynyl)phenyl)ethynyl)phenyl)acetamide ((*S*)-**3** or (*R*)-**3**, 0.42 g, 0.74 mmol, 1.00 equiv.) was dissolved in THF (10 mL). Next TBAF was added (1M in hexane, 0.89 mL, 0.89 mmol, 1.20 equiv.) and the reaction was stirred at r.t. for 15 minutes. The crude was chromatographed on silica gel (70-230 mesh) with hexane/ethyl acetate (70:30) as eluent (0.45 g, 88% of yield).

^{1}H NMR (300 MHz, $CDCl_3$) δ_H (ppm): 8.62 (s, 1H), 7.60 (d, 2H), 7.53-7.34 (m, 15H), 4.75 (s, 1H), 3.47 (s, 3H), 3.18 (s, 1H).

^{13}C NMR (75 MHz, $CDCl_3$) δ_C (ppm): 168.5, 140.0, 137.5, 136.3, 132.4, 132.1, 131.5, 131.5, 128.7, 127.0, 123.5, 123.5, 122.6, 122.0, 119.4, 118.7, 100.3, 91.2, 91.1, 90.6, 88.8, 83.8, 83.2, 79.0, 57.4.

HRMS (ESI) m/z calcd for $C_{33}H_{24}NO_2$ $[M+H]^+$: 466.1829, found: 466.1802.

For m-(*S*)-**3**, $[a]_D^{20} =$ +17.80 (c = 5 mg·mL^{-1}, DCM)

For m-(*R*)-**3**, $[a]_D^{20} =$ -15.14 (c = 5 mg·mL^{-1}, DCM)

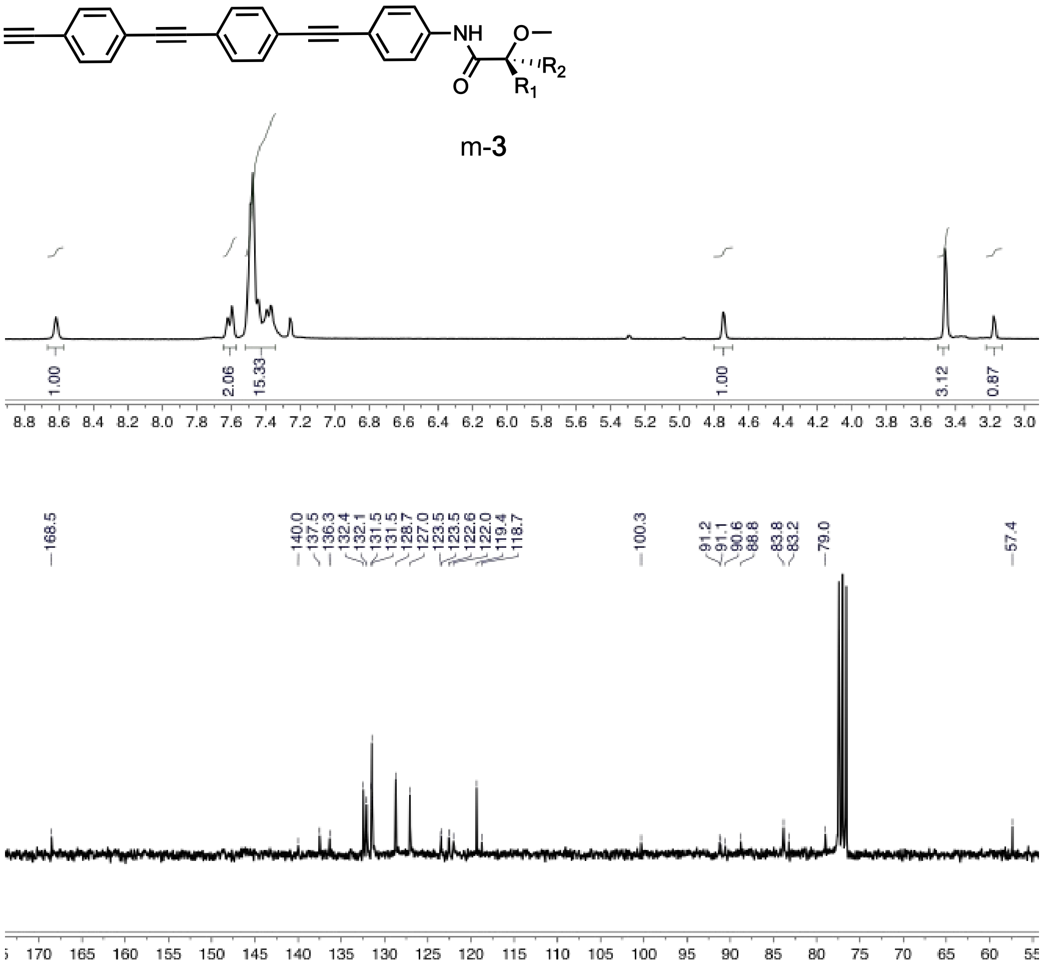

Figure S6. ^{1}H-NMR and ^{13}C-NMR of m-**3** in $CDCl_3$.

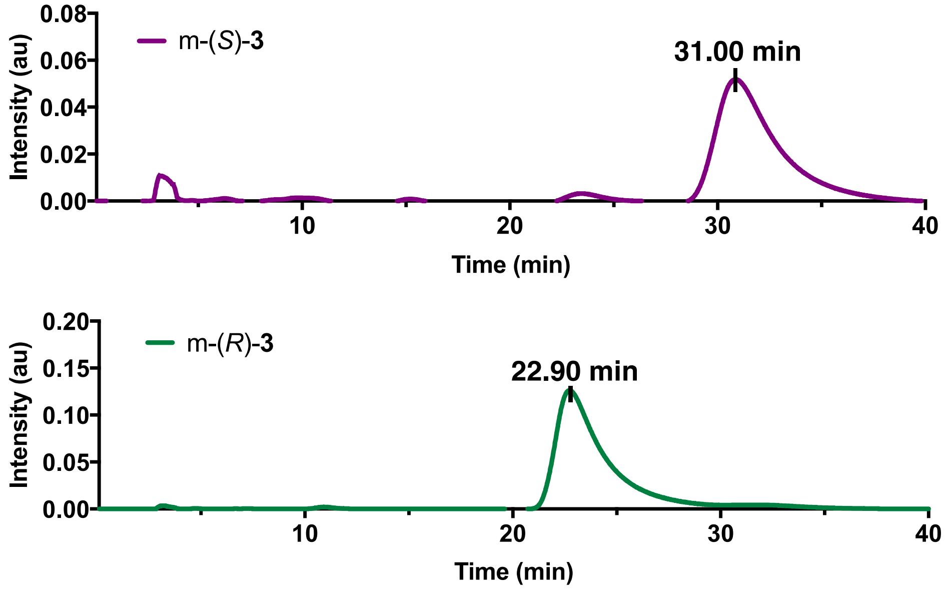

Figure S7. Chiral HPLC traces for m-(*S*)-**2** and m-(*R*)-**2** respectively.

3. Crystallographic Data

A summary of the crystallographic results is given bellow: Crystals of m-(*R*)-**2** and m-(*S*)-**3** for X-ray analysis were grown by slow evaporation from a 2:8 mixture of DCM/Hexane. A crystal was selected and mounted on a MiTeGen loop under oil and flash frozen at 100 K under a cold N_2 gas stream. X-ray diffraction data were collected on a Bruker X8 kappa APEX II CCD diffractometer.

Data collection: Bruker APEX2 software, [SAINT (version 8.34A), SADABS (version 2014/5), BrukerAXS Inc, Madison, Wisconsin, USA.)]; cell refinement: SAINT V8.34A integration software; absorption correction: SADABS2014/5 (Bruker (2014) APEX2 (Version 2014.11-0); data reduction: SORTAV3; program(s) used to solve structure: SHELXD2013/24 ; program used to refine structure: SHELXL2014/75 ; molecular graphics: ORTEP-3 for Windows 6 ; software used to prepare material for publication: WinGX publication routines.

Crystal data for m-(*R*)-**2**

Crystal data

$C_{25}H_{19}NO_2$
M_r = 365.41
Orthorhombic, $P2_12_12_1$
a = 5.2753 (2) Å
b = 8.2287 (2) Å
c = 45.0822 (11) Å
V = 1956.97 (10) Å^3
Z = 4
F(000) = 768

D_x = 1.24 Mg m^{-3}
Mo $K\alpha$ radiation, λ = 0.71073 Å
Cell parameters from 7950 reflections
θ = 2.5–27.0°
μ = 0.08 mm^{-1}
T = 100 K
Needle, colourless
0.52 × 0.09 × 0.05 mm

Data collection

Bruker APEX-II diffractometer
Radiation source: sealed x-ray tube
Graphite monochromator
φ or ω oscillation scans
Absorption correction: multi-scan *SADABS2014*/5 - Bruker AXS area detector scaling and absorption correction
T_{min} = 0.905, T_{max} = 0.991

28768 measured reflections
4486 independent reflections
3752 reflections with $I > 2\sigma(I)$
R_{int} = 0.041
θ_{max} = 27.5°, θ_{min} = 1.8°
$h = -6 \rightarrow 6$
$k = -10 \rightarrow 10$
$l = -57 \rightarrow 58$

Refinement

Refinement on F^2
Least-squares matrix: full
$R[F^2 > 2\sigma(F^2)]$ = 0.041
$wR(F^2)$ = 0.086
S = 1.04
4486 reflections
258 parameters
0 restraints
0 constraints
Primary atom site location: other
Secondary atom site location: difference Fourier map
Hydrogen site location: mixed

H atoms treated by a mixture of independent and constrained refinement
$w = 1/[\sigma^2(F_o^2) + (0.0379P)^2 + 0.3837P]$ where $P = (F_o^2 + 2F_c^2)/3$
$(\Delta/\sigma)_{max} < 0.001$
$\Delta\rho_{max}$ = 0.19 e Å^{-3}
$\Delta\rho_{min}$ = −0.20 e Å^{-3}
Absolute structure: Flack x determined using 1346 quotients [(I+)-(I-)]/[(I+)+(I-)] (Parsons, Flack and Wagner, Acta Cryst. B69 (2013) 249-259).
Absolute structure parameter: −0.2 (5)

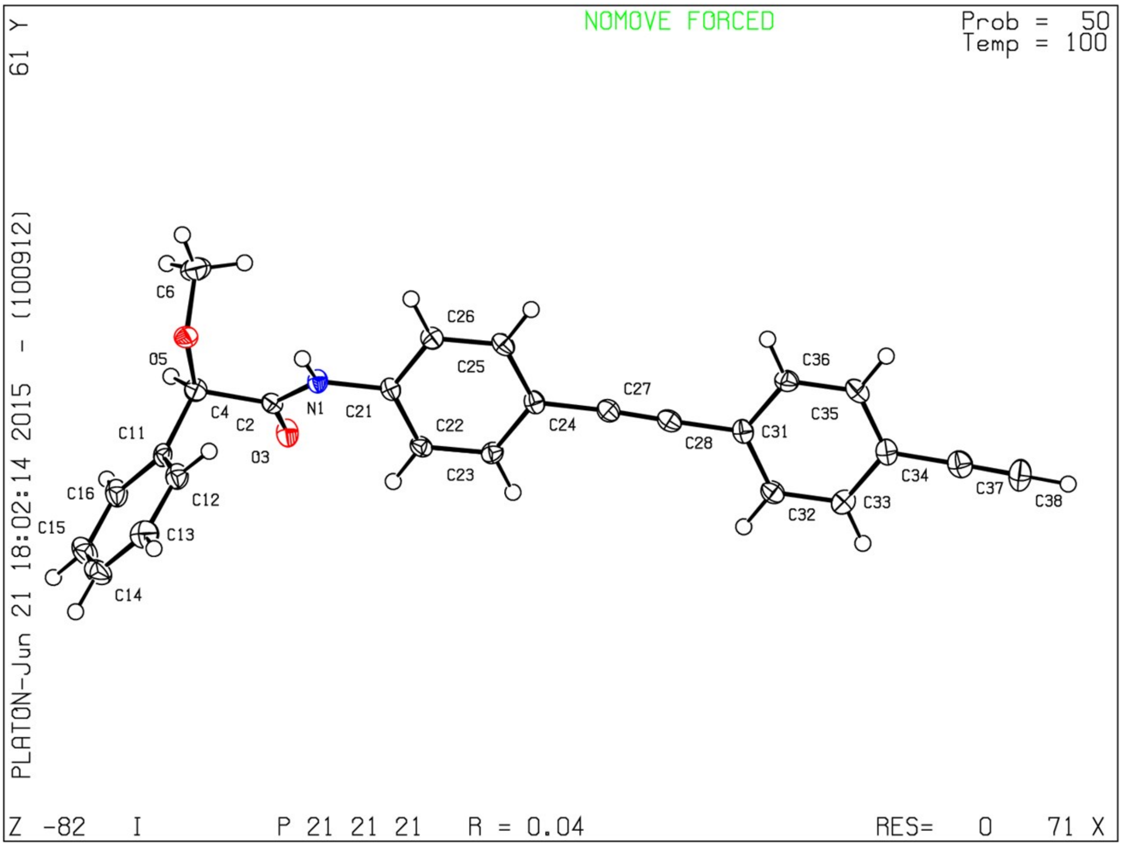

Figure S8. Crystal data and ORTEP view of the asymmetric unit content for m-(*R*)-**2**

Crystal data for m-(*S*)-**3**

Crystal data

$C_{33}H_{23}NO_2$
M_r = 465.52
Orthorhombic, $P2_12_12_1$
Hall symbol: P 2ac 2ab
a = 5.3349 (2) Å
b = 8.0996 (4) Å
c = 58.179 (2) Å
V = 2513.94 (18) Å^3
Z = 4
$F(000)$ = 976
D_x = 1.23 Mg m^{-3}
Cu $K\alpha$ radiation, λ = 1.54184 Å
Cell parameters from 1556 reflections
θ = 3.0–74.1°
μ = 0.60 mm^{-1}
T = 100 K
Plate, colourless
0.09 × 0.08 × 0.02 mm

Data collection

Bruker D8 VENTURE PHOTON III-14 diffractometer
Radiation source: INCOATEC microfocus sealed tube, Incoatec IμS 3.0
Incoatec multilayer mirror monochromator
Detector resolution: 7.3910 pixels mm^{-1}
ω and phi scans
Absorption correction: multi-scan BRUKER *SADABS2016*/2
T_{min} = 0.798, T_{max} = 0.971
22404 measured reflections
5141 independent reflections
2954 reflections with $I > 2\sigma(I)$
R_{int} = 0.138
θ_{max} = 74.8°, θ_{min} = 3.0°
$h = -6 \rightarrow 4$
$k = -10 \rightarrow 10$
$l = -72 \rightarrow 70$

Refinement

Refinement on F^2
Least-squares matrix: full
$R[F^2 > 2\sigma(F^2)]$ = 0.076
$wR(F^2)$ = 0.205
S = 1.00
5141 reflections
334 parameters
0 restraints
0 constraints
Primary atom site location: dual
Secondary atom site location: dual
Hydrogen site location: mixed
H atoms treated by a mixture of independent and constrained refinement
$w = 1/[\sigma^2(F_o^2) + (0.092P)^2]$ where $P = (F_o^2 + 2F_c^2)/3$
$(\Delta/\sigma)_{max}$ < 0.001
$\Delta\rho_{max}$ = 0.31 e Å^{-3}
$\Delta\rho_{min}$ = −0.29 e Å^{-3}
Absolute structure: Flack x determined using 792 quotients [(I+)-(I-)]/[(I+)+(I-)] (Parsons, Flack and Wagner, Acta Cryst. B69 (2013) 249-259).
Absolute structure parameter: 0.0 (6)

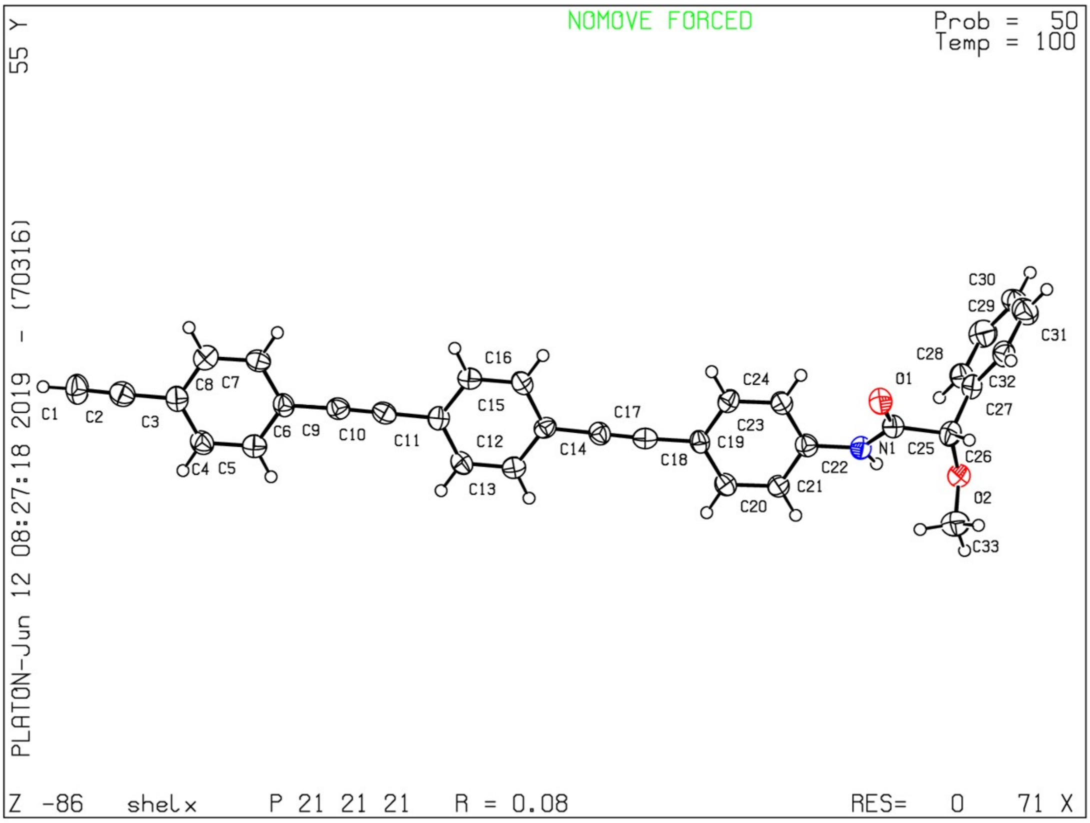

Figure S9. Crystal data and ORTEP view of the asymmetric unit content for m-(*S*)-**3**

4. Theoretical Calculations for Monomers

TD-DFT(CAM-B3LYP) calculations were performed, using the 3-21G basis set, for m-(*R*)-**2** and m-(*S*)-**3**. The data obtained are in good agreement with the experimental information obtained for the aforementioned monomers. This confirms the *ap* conformation adopted by the pendant.

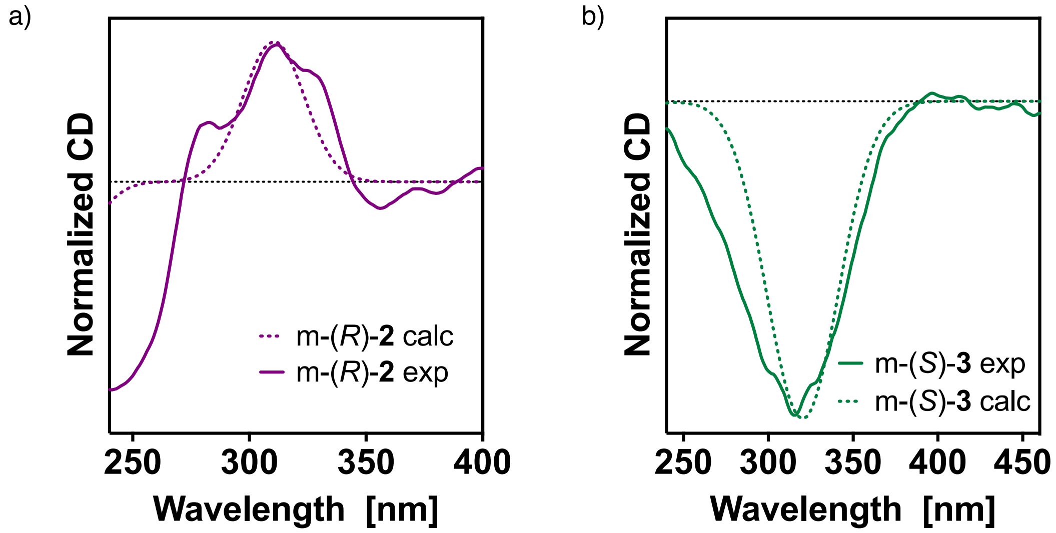

Figure S10. Comparison of the calculated and experimental CD spectra obtained for (a) m-(*R*)-**2** and (b) m-(*S*)-**3** (Full width at half-maximum (FWHM) equals 20 nm).

5. Synthesis of Polymers

5.1 General Procedure for Polymerization

The polymers were synthesized in a flask (sealed ampoule) previously dried under vacuum and flushed with Ar for three times. The monomers were added as a solid and dissolved in dry THF. Next a solution of rhodium norbornadiene chloride dimer $[Rh(nbd)Cl]_2$ was added and the mixture was stirred overnight at 20 ºC. The resulting polymers were diluted in CH_2Cl_2 and precipitated in a large amount of MeOH, centrifuged and reprecipitated in hexane and centrifuged again.

Table S1. Calculated amounts for the synthesis of the polymers.

Monomer	Mass (mg)	THF (μL)	Et_3N (μL)	Catalyst (mg)	Yield (%)
m-(*S*)-**2**	50	400	5	0.6	70
m-(*R*)-**2**	50	400	5	0.6	80
m-(*S*)-**3**	50	500	5	0.5	72
m-(*R*)-**3**	50	500	5	0.5	77

5.2 Low Temperature Polymerization

The general procedure was followed. Before the addition of the catalyst the corresponding monomer solution was cooled to 0 ºC. The polymerization was allowed to reach to room temperature and it was stirred overnight.

5.3 NMR and Raman Experiments

The *cis* configuration of the polyenic backbone was determined by ^{1}H NMR spectroscopy (vinylic protons resonate at δ = 5.7-5.8 ppm). The bands observed by Raman resonance confirmed the former configuration. The peak at highest wavelength corresponds to the C=C bond stretching and overlaps with that of the phenyl ring. The band at 1350-1340 cm^{-1} arises from the *cis* C-C bond coupled with the single bond connecting the main chain and the phenyl ring. The peak at lowest wavelength corresponds to the C-H bond of the *cis* form. The disappearance of the alkyne peak (ca. 2110 cm^{-1}) also confirms the formation of the polymer.

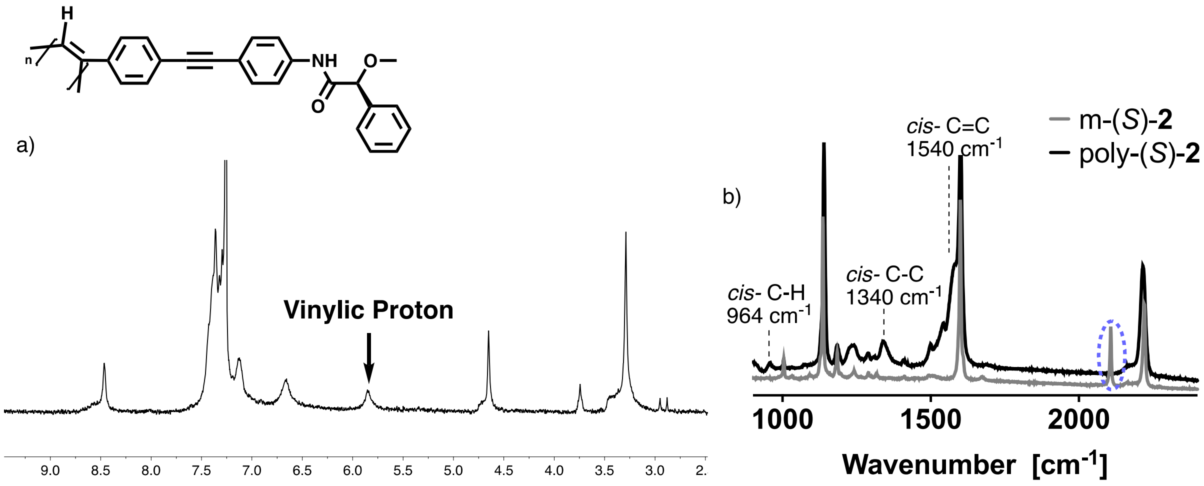

Figure S11. (a) ^{1}H-NMR for poly-**2** in $CDCl_3$ and (b) Raman spectra comparison.

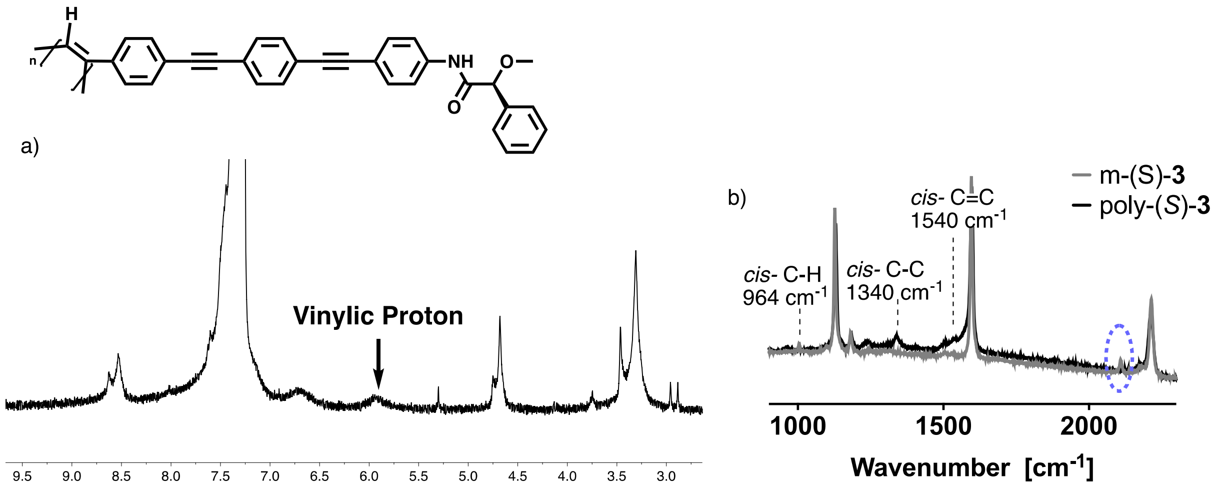

Figure S12. (a) ^{1}H-NMR for poly-**3** in $CDCl_3$ and (b) Raman spectra comparison.

6. GPC Studies

The molecular weight was estimated by GPC.

Table S2. GPC data for poly-**2**, poly-**3** and poly-**3** at 0 ºC.

Polymer	Mn	Mw	Mz	PDI
Poly-**2**	22802	26941	33147	1.2
Poly-**3**	25383	67530	213386	2.7
Poly-**3** 0 ºC	28286	60234	201811	2,1

7. Thermal Studies

7.1 DSC Studies

The geometry of the polymer was determined by DSC. As a general protocol, the polymer was placed in an aluminum pan up to 400 ºC (heating rate: 10 ºC min^{-1}). The thermograms for poly-(*S*)-**2** and poly-(*S*)-**3** show a typical *c-c* trace with an exothermal peak, at 210 °C and 287 °C respectively, correspondent to a *t-t* transition. It is observed that the geometry of the polymer is kept despite the introduction of the spacer.

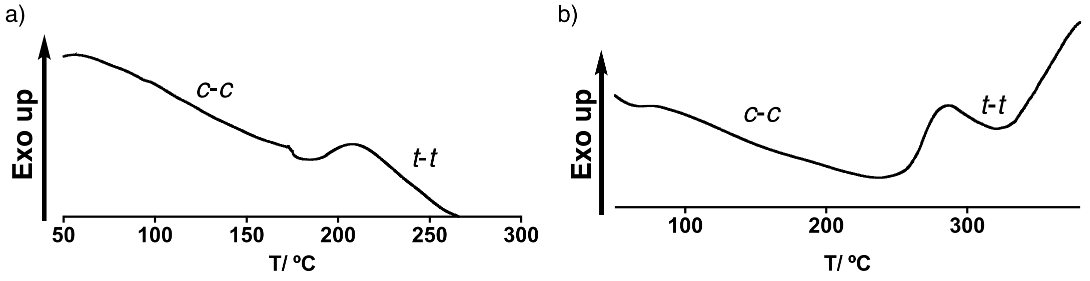

Figure S13. DSC studies for (a) poly-(*S*)-**2** and (b) poly-(*S*)-**3**.

7.2 TGA Studies

The thermal stability was evaluated by TGA. As a general protocol, the polymer sample was placed in a platinum pan and heated from 40°C to 800°C (heating rate: 10 °C min^{-1}). Poly-(*S*)-**2** starts to degrade at 300 °C while poly-(*S*)-**3** starts at 370 °C.

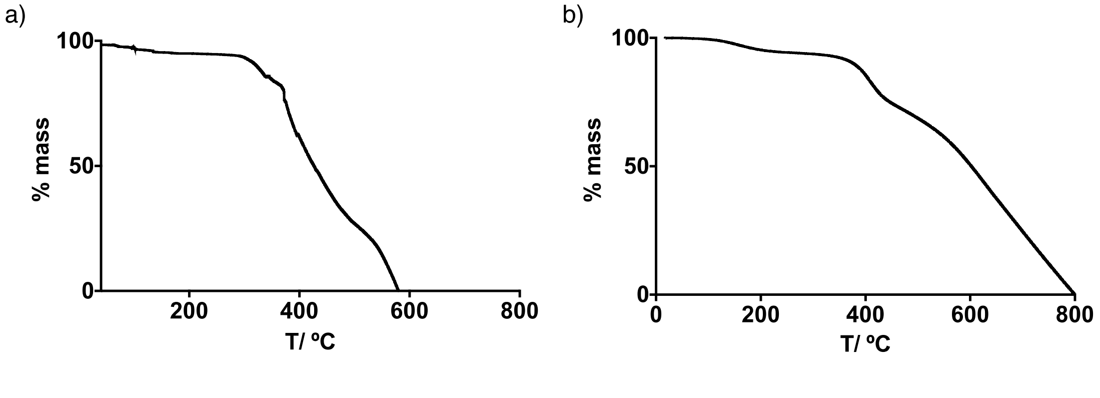

Figure S14. TGA thermograms (a) poly-(*S*)-**2** and (b) poly-(*S*)-**3**.

8. Conformational Studies

8.1 Solution Studies

Poly-(*S*)-**2** and poly-(*S*)-**3** (0.2 mg mL^{-1}) were tested in different solvents. Poly-(*S*)-**2** shows a better folding in polar solvents, whereas poly-(*S*)-**3** exhibits almost the same CD intensity in all cases.

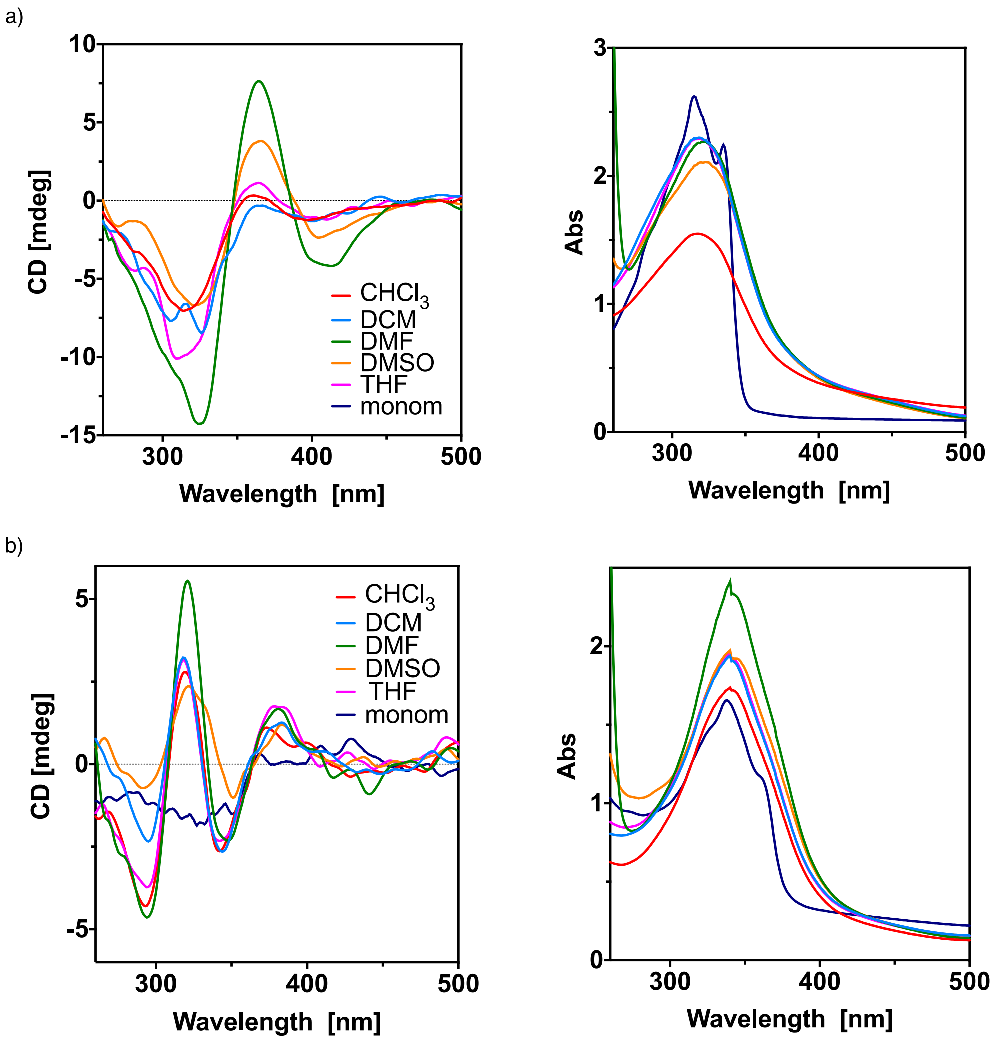

Figure S15. CD and UV-Vis spectra for (a) poly-(S)-**2** and (b) poly-(S)-**3** in different solvents.

8.2 Response to External Stimuli: Interaction with Metals

Both polymers were titrated with different divalent and monovalent perchlorates. Poly-(*S*)-**2** and poly-(*S*)-**3** were dissolved in $CHCl_3$ (0.1 mg mL^{-1} and 0.2 mg mL^{-1} respectively) and the correspondent metal salt solution (THF, 50 mg mL^{-1}) was then added.

Poly-(*S*)-**2** shows a chiral enhancement for the case of Li^+, Ag^+, Na^+ (Figure S16a, b and c) and Ba^{2+} (Figure S17a). Interestingly for Ca^{2+}, a gradual inversion is observed in the monomeric region accompanied with a disappearance of the vinylic band (Figure S17b).

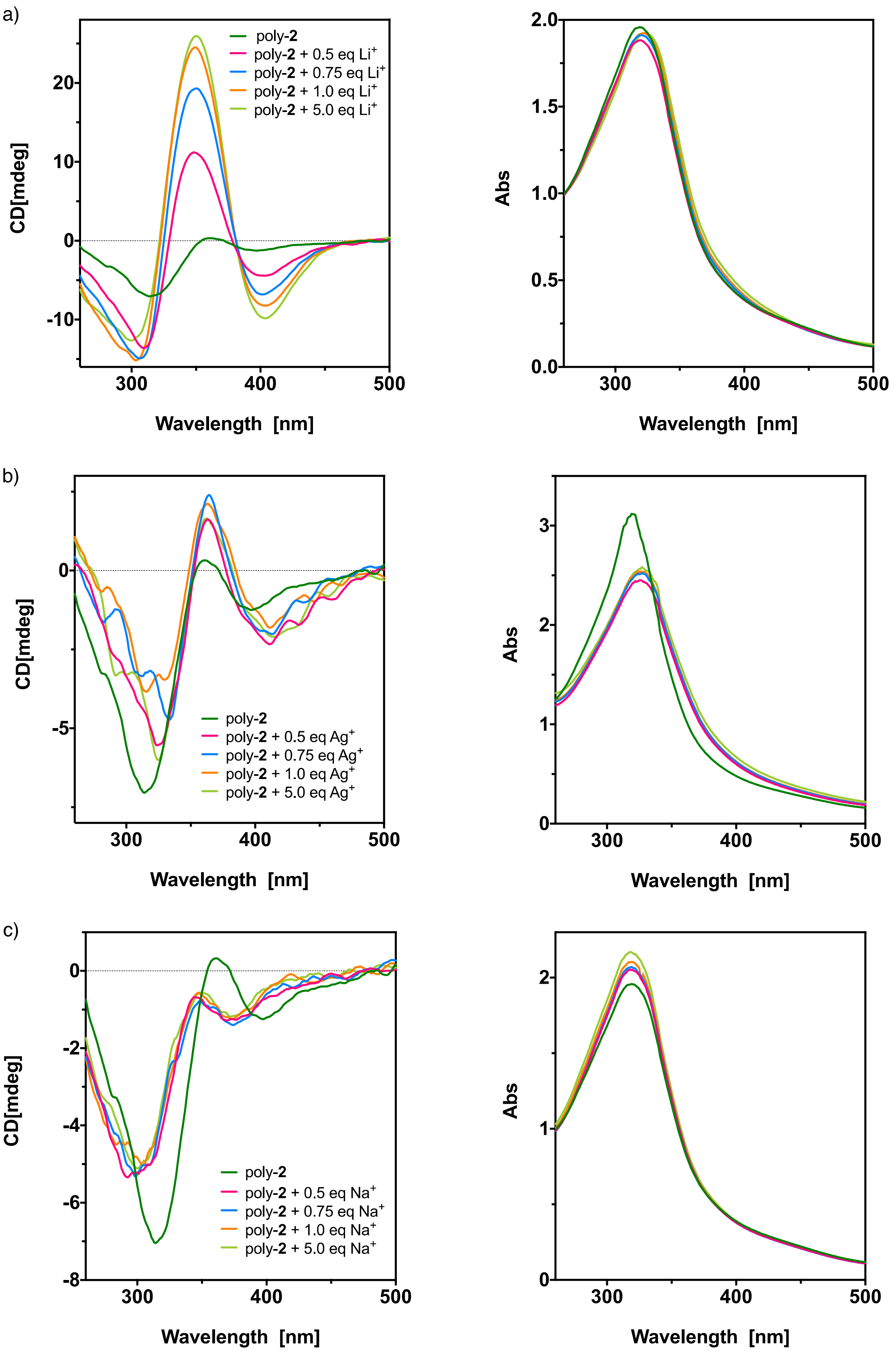

Figure S16. CD and UV-Vis spectra for poly-**2** ($CHCl_3$, 0.1 mg mL^{-1}) titrated with different amounts of (a) $LiClO_4$, (b) $AgClO_4$ and (c) $NaClO_4$ (THF, 50 mg mL^{-1}).

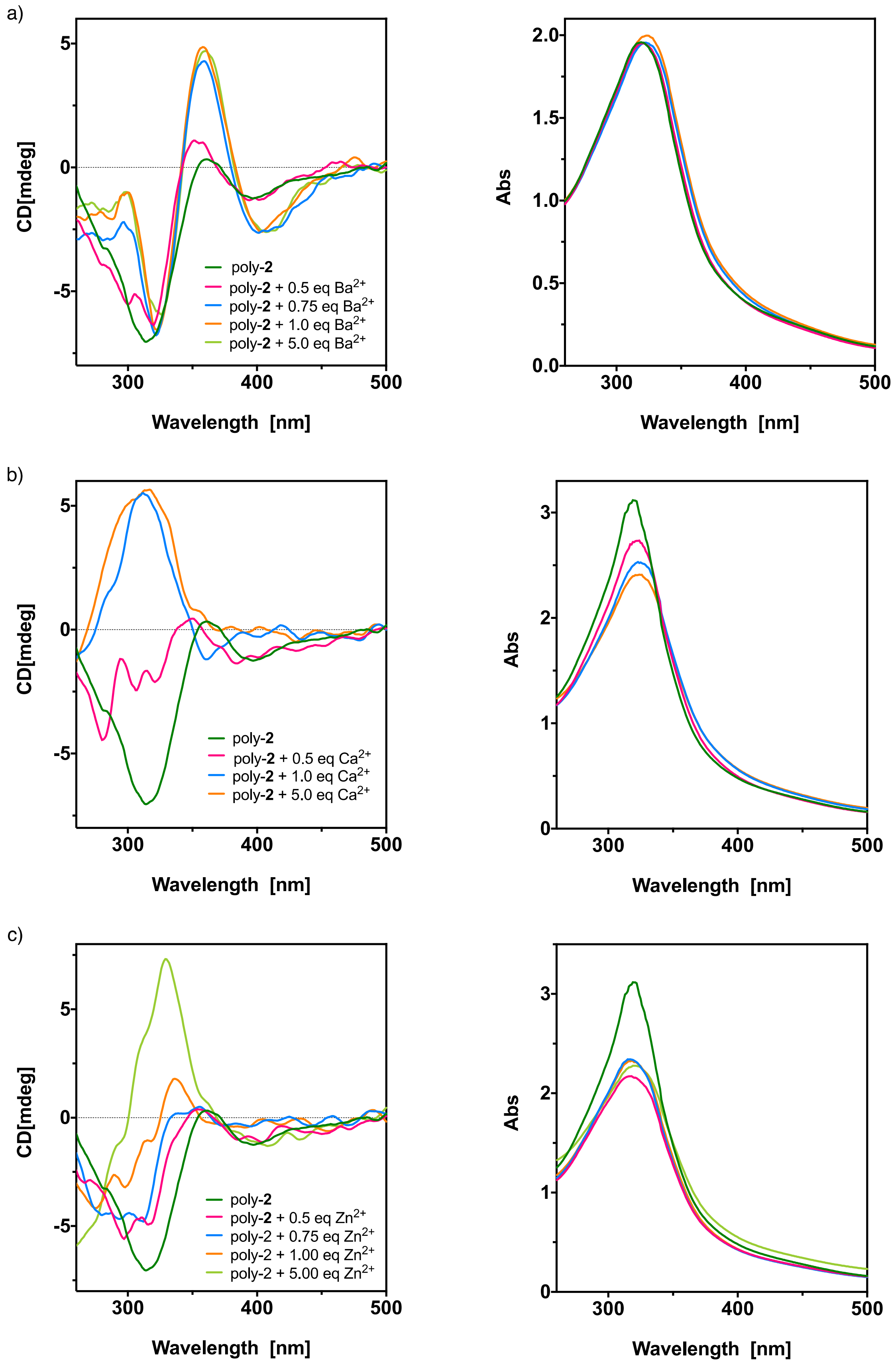

Figure S17. CD and UV-Vis spectra for poly-**2** ($CHCl_3$, 0.1 mg mL^{-1}) titrated with different amounts of (a) $Ba(ClO_4)_2$ (b) $Ca(ClO_4)_2$ and (c) $Zn(ClO_4)_2$ (THF, 50 mg mL^{-1}).

Similar experiments were performed for poly-**3**. Neither amplification of chirality nor inversion were observed.

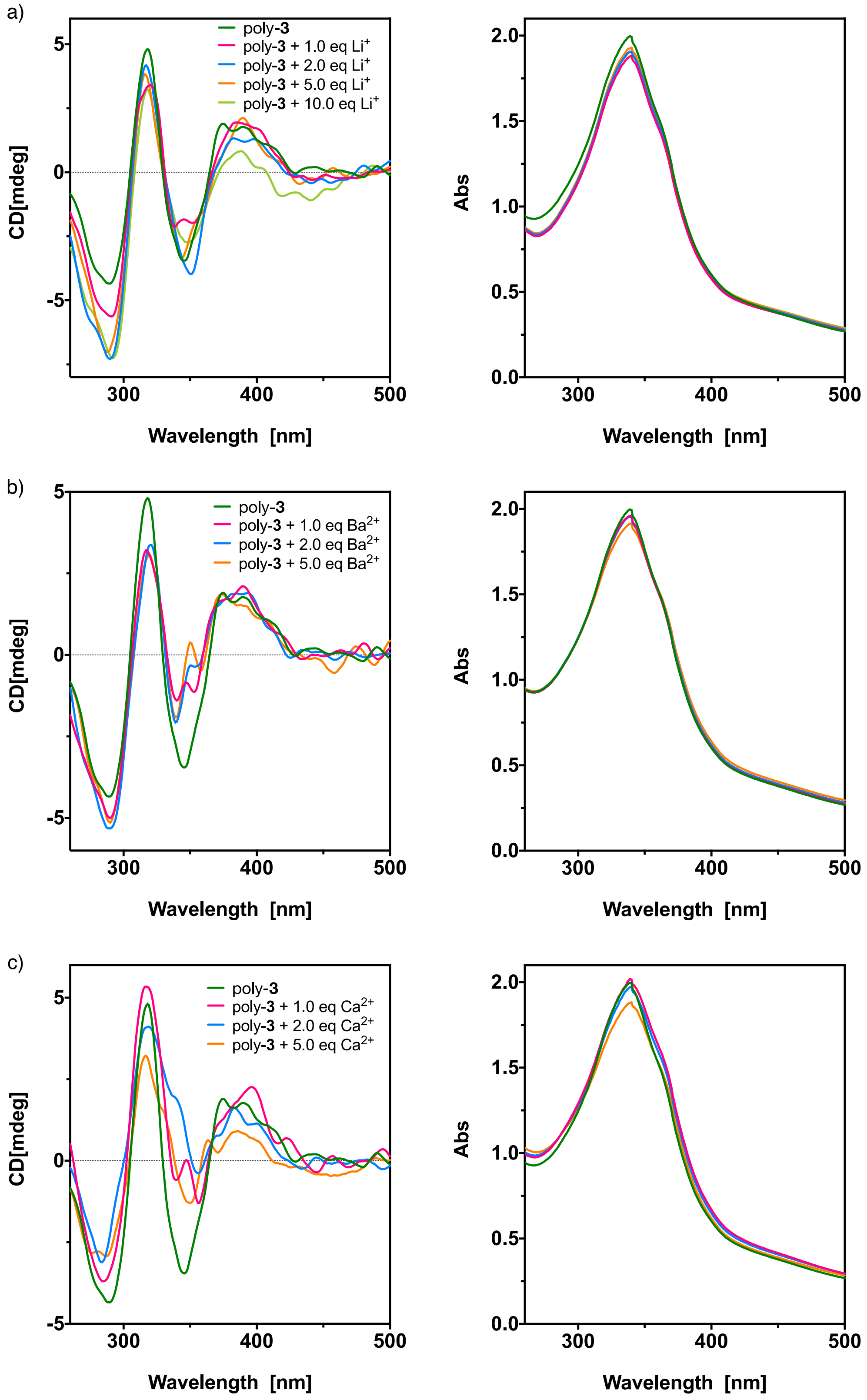

Figure S18. CD and UV-Vis spectra for poly-**3** ($CHCl_3$, 0.2 mg mL^{-1}) titrated with different amounts of (a) $LiClO_4$ and (b) $BaClO_4$ and (c) $Ca(ClO_4)_2$ (THF, 50 mg mL^{-1}).

9. ATR/FT-IR Studies

$M(ClO_4)_2$ or $MClO_4$ (THF, 50 mg mL^{-1}) was added to a solution of poly-**2** ($CHCl_3$, 3 mg mL^{-1}). The mixture was allowed to react for five minutes and analyzed by FT-IR.

In all cases, an increase of the degree of association is observed only for the carbonyl group —*ap* conformation—, except for Ca^{2+}, in which the degree of association is also increased in the methoxy group —*sp* conformation—. This is in full agreement with the CD studies.

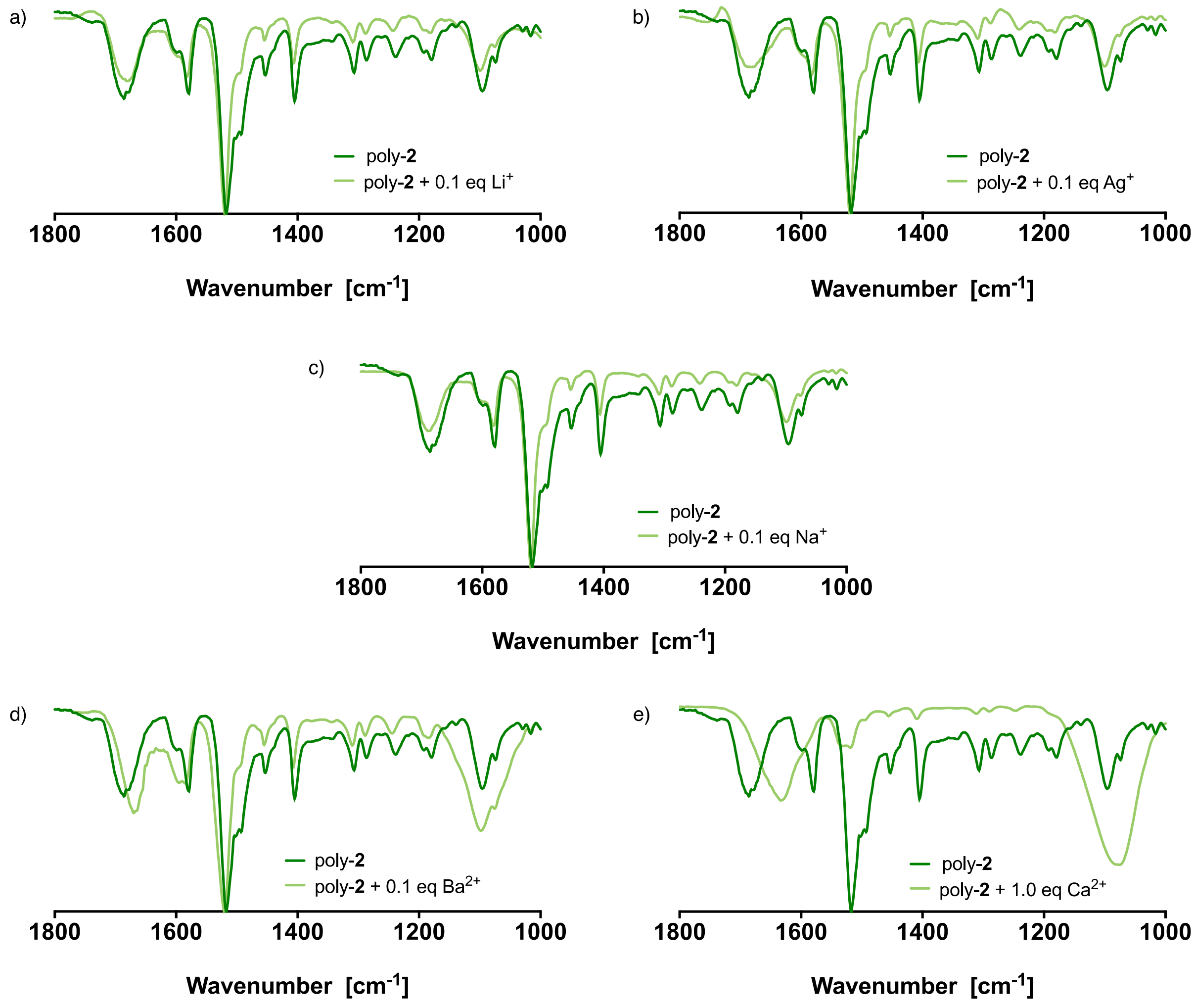

Figure S19. IR spectra of a solution of poly-**2** in $CHCl_3$ (3 mg mL^{-1}) titrated with (a) $LiClO_4$ (THF, 50 mg mL^{-1}), (b) $AgClO_4$ (THF, 50 mg mL^{-1}), (c) $NaClO_4$ (THF, 50 mg mL^{-1}), (d) $Ba(ClO_4)_2$ (THF, 50 mg mL^{-1}) and (e) $Ca(ClO_4)_2$ (THF, 50 mg mL^{-1}).

Table S3. FT-IR data of poly-**2** in solution.

	υ_{CO}	υ_{Ome}	$\Delta\upsilon_{CO}$	$\Delta\upsilon_{Ome}$
Poly-**2**	1685	1091	–	–
Poly-**2** + 0.1 eq Li^+	1675	1096	10	- 5
Poly-**2** + 0.1 eq Na^+	1679	1097	6	- 6
Poly-**2** + 0.1 eq Ag^+	1664	1094	21	- 3
Poly-**2** + 0.1 eq Ba^{2+}	1675	1096	10	- 5
Poly-**2** + 1.0 eq Ca^{2+}	1627	1070	58	-21

10. Cation-π Experiments

A solution of poly-**2** in $CDCl_3$ (3 mg mL^{-1}) was titrated with 2.0 equivalents of $LiClO_4$ (10 mg mL^{-1} in CD_3OD) and studied by ^7Li NMR spectroscopy. The shielding in the ^7Li chemical shift is indicative of a cation-π interaction, related to an *ap* conformation of the pendant group. This is in accordance with the information obtained from the CD and FT-IR experiments.

12. Low Temperature Polymerization

It was observed that when m-(*S*)-**3** is polymerized at low temperature, the resulting polymer presents a helical enhancement when compared to the polymer obtained at 20 ºC. At 0 ºC, the polymerization mechanism takes place at a lower rate, allowing the preorganization of the monomer and resulting in a better-folded polymer.

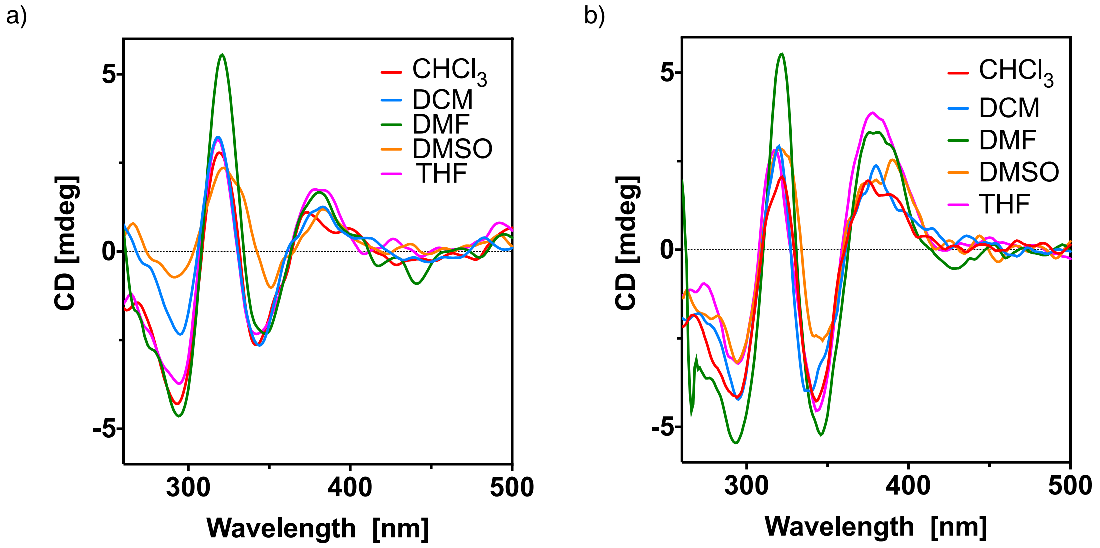

Figure S20. CD trace for poly-(*S*)-**3** in different solvents polymerized at (a) 25 ºC and (b) 0 ºC.

In order to get a better comparison between the CD traces, the data was normalized and the spectra was represented into the molar value.

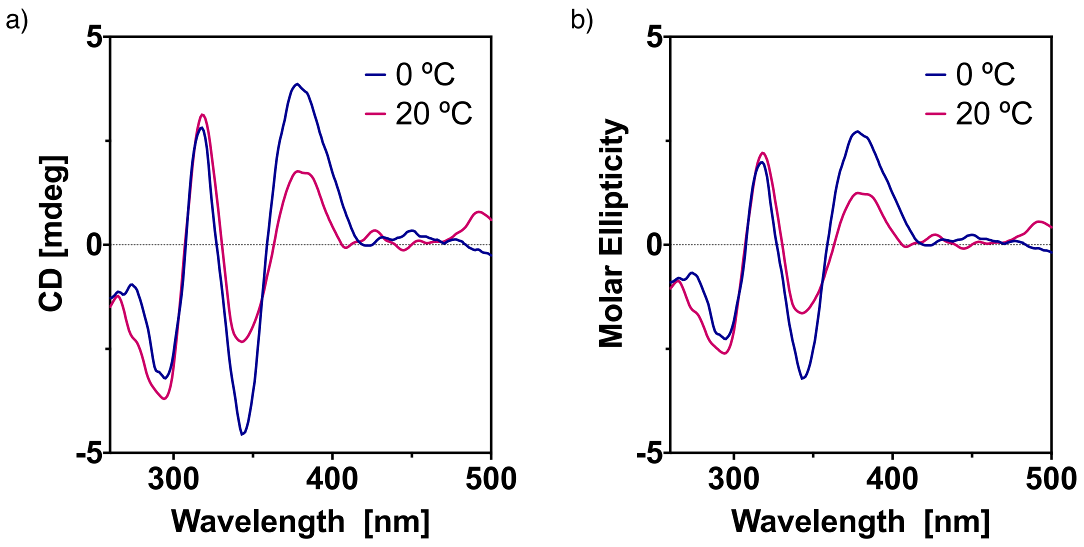

Figure S21. CD traces in THF polymerized at different temperatures and represented in (a) mdeg and (b) molar ellipticity.

11. VT-CD Experiments

Poly-(*S*)-**2** (0.2 mg mL^{-1}, DMF) shows a decrease in the CD intensity when increasing the temperature. On the other hand, the CD trace of poly-(*S*)-**3** (0.2 mg mL^{-1}, DMF) is slightly diminished when changing the temperature.

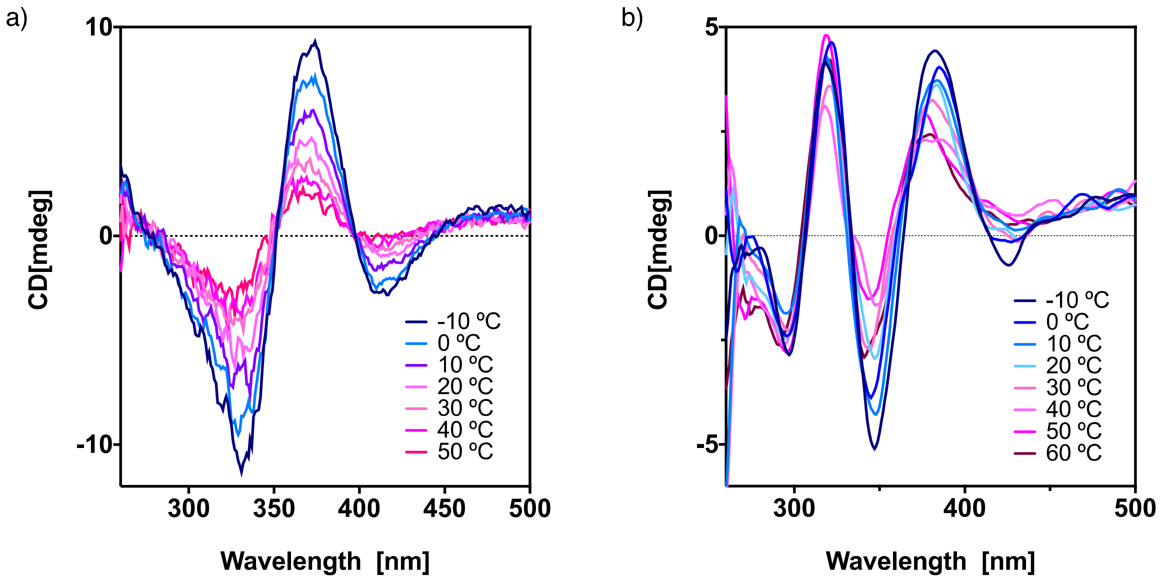

Figure S22. VT-CD of (a) poly-**2** and (b) poly-**3** heating rate 10 ºC min^{-1}.

12. Atomic Force Microscopy (AFM) Measurements

a)

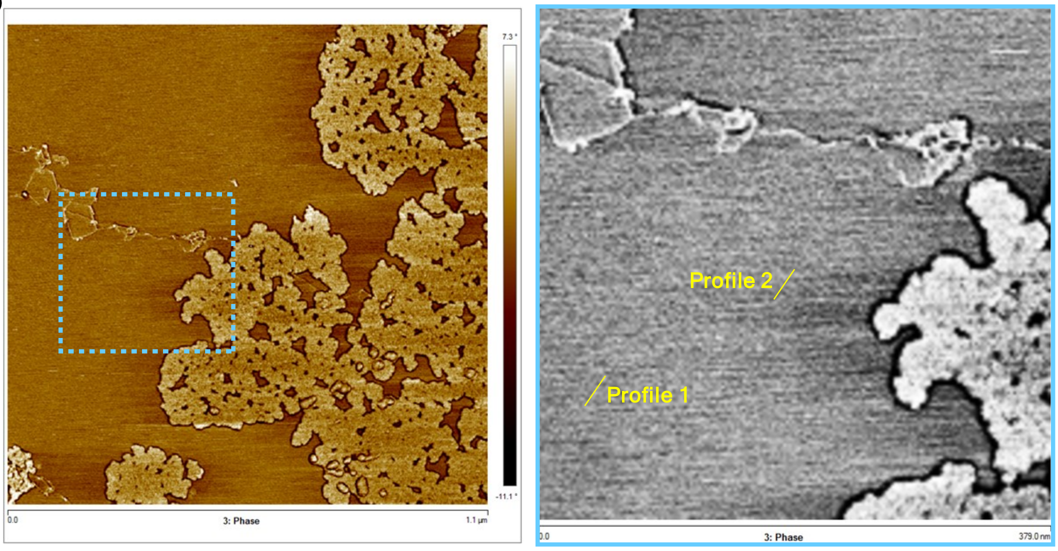

b)

Average chain separation measured on the indicated profiles = 5.6 nm

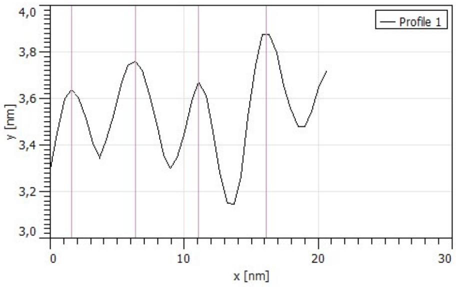

Points	X [nm]	Y [pm]	Length [nm]
	1,59	3643	
	6,36	3761	4,77
	11,06	3670	4,70
	16,12	3879	5,06

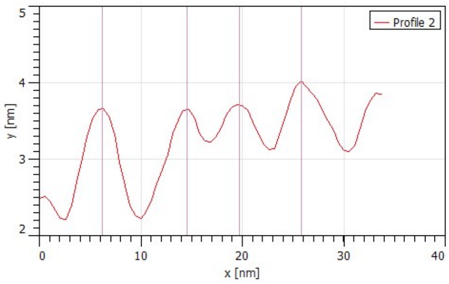

Points	X [nm]	Y [nm]	Length [nm]
	6,20	3,676	
	14,55	3,656	8,36
	19,72	3,718	5,16
	25,82	4,038	6,10

c)

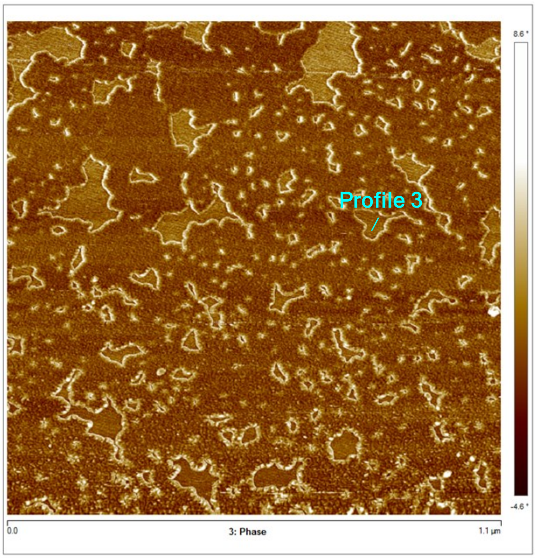

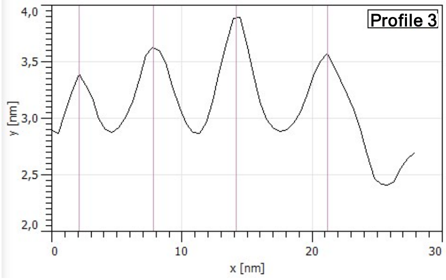

Points	X [nm]	Y [nm]	Length [nm]
	2,10	3,395	
	7,81	3,632	5,71
	14,17	3,896	6,36
	21,18	3,578	7,01

Figure S23. (a) AFM phase images for poly-(*S*)-**2**, from large scale image to magnification of the highlighted area. (b) Graphics showing the chain distribution profiles measured in the indicated zone. (c) Additional AFM phase image and the corresponding chain separation distribution.

a)

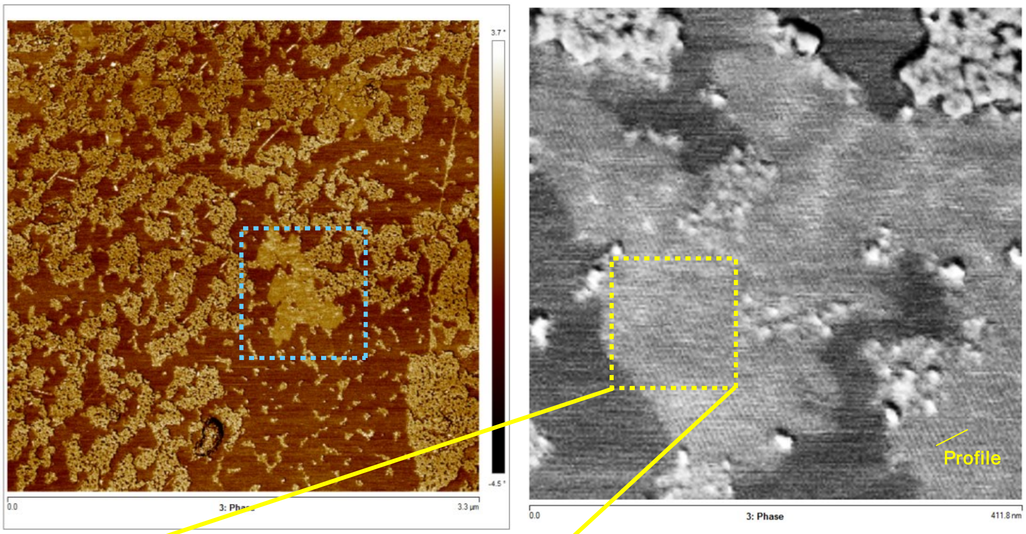

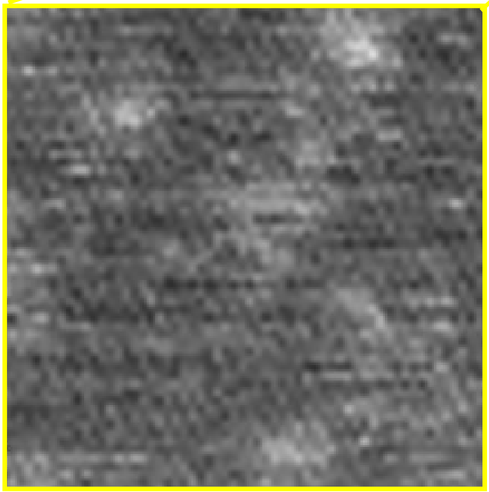

Points	X [nm]	Y [nm]	Length [nm]
	1,13	4,721	
	5,14	4,961	4,01
	9,72	4,818	4,58
	14,08	4,509	4,37
	17,46	4,674	3,38
	22,04	4,566	4,58
	26,55	4,594	4,51

Average chain separation = 4.2 nm

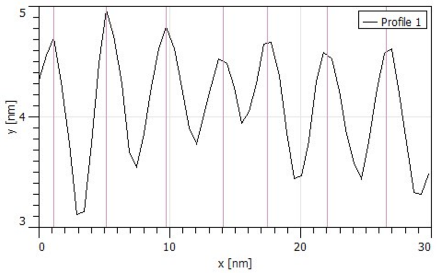

b)

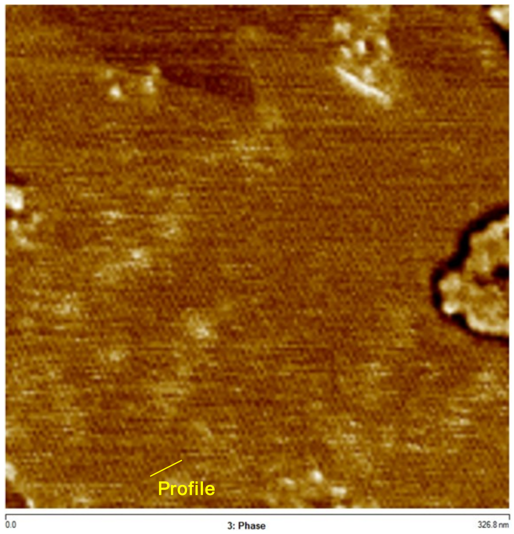

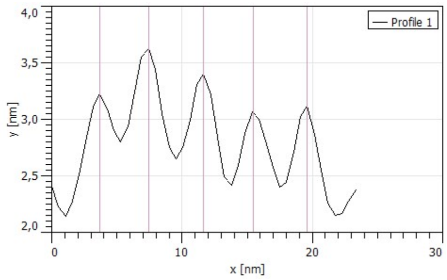

Points	X [nm]	Y [nm]	Length [nm]
	3,69	3,224	
	7,45	3,629	3,76
	11,64	3,403	4,19
	15,47	3,062	3,83
	19,59	3,119	4,12

Figure S24. (a) AFM phase images for poly-(*S*)-**3**. From large scale images to magnification of the highlighted area showing the helical chains in detail. Graphic showing the chain distribution profile measured in the indicated zone. (b) Additional AFM phase image and the corresponding chain separation distribution.

13. Theoretical Calculations for Polymers

13.1 Computational Details

ECD spectroscopy is a powerful tool for the study of the absolute configuration and conformation of optically active molecules. From the computational point of view, the main quantity to be evaluated is the scalar rotational strength, that for a transition from the ground state of the molecule (0) to an excited state (*n*), can be calculated from the residue of the linear response function corresponding to the electric dipole-magnetic dipole polarizability tensor (R_{0n}^{LG}). In the length-gauge (LG) formalism, the residue is given by equation (1):

$$R_{0n}^{LG} = \sum_{\alpha} \lim_{\omega \to \omega_{0n}} \left(\omega - \omega_{0n}\right) \ll \mu_\alpha; m_\alpha \gg_\omega \qquad (1)$$

Where ω_{0n} is the frequency of the transition between 0 and the *n* state, μ_α is the α component of the electric dipole moment and m_α is the α component of the magnetic dipole moment of the molecule.[S2]

Considering the difficulties to carry out ECD theoretical calculations on large polymers, representative oligomers will be used —n = 9, where n denotes the number of monomer repeating units (m.r.u.)—.

The input structures used for ECD calculations were adjusted to the experimental data obtained from structural techniques such as DSC, AFM and UV-Vis spectroscopy, defining the four different dihedral angles needed to build up the helical scaffold (ω_1, ω_2, ω_3 and ω_4). Moreover, the pendant groups were introduced in *ap* conformation —confirmed by experimental studies—. 3-D structures of the oligomers for poly-**2** and poly-**3** were obtained and submitted for ECD calculations (Figure S25).

To evaluate the theoretical ECD time dependent density functional theory (TD-DFT)[S3] calculations, with the CAM-B3LYP functional[S4] and the 3-21G basis set,[S5] were carried out for the aforementioned oligomers using the ORCA program (80 excitations were included).[S6] The Gabedit[S7] code was used to plot the spectra. The obtained theoretical ECD spectra for these oligomers were compared to the experimental ones, observing that the results are in good agreement. For an efficient comparison and taking into account the tendency of the TD-DFT method to overestimate the excitation energies, the wavelength and intensity at the maximum/minimum corresponding to the first Cotton effect of the theoretical spectra were adjusted to the experimental spectra. Employing the same correction

factors, the lambdas were shifted and the intensities rescaled (vide infra). In all cases the agreement was reasonable after the above-mentioned correction has been carried out (Figure S25).

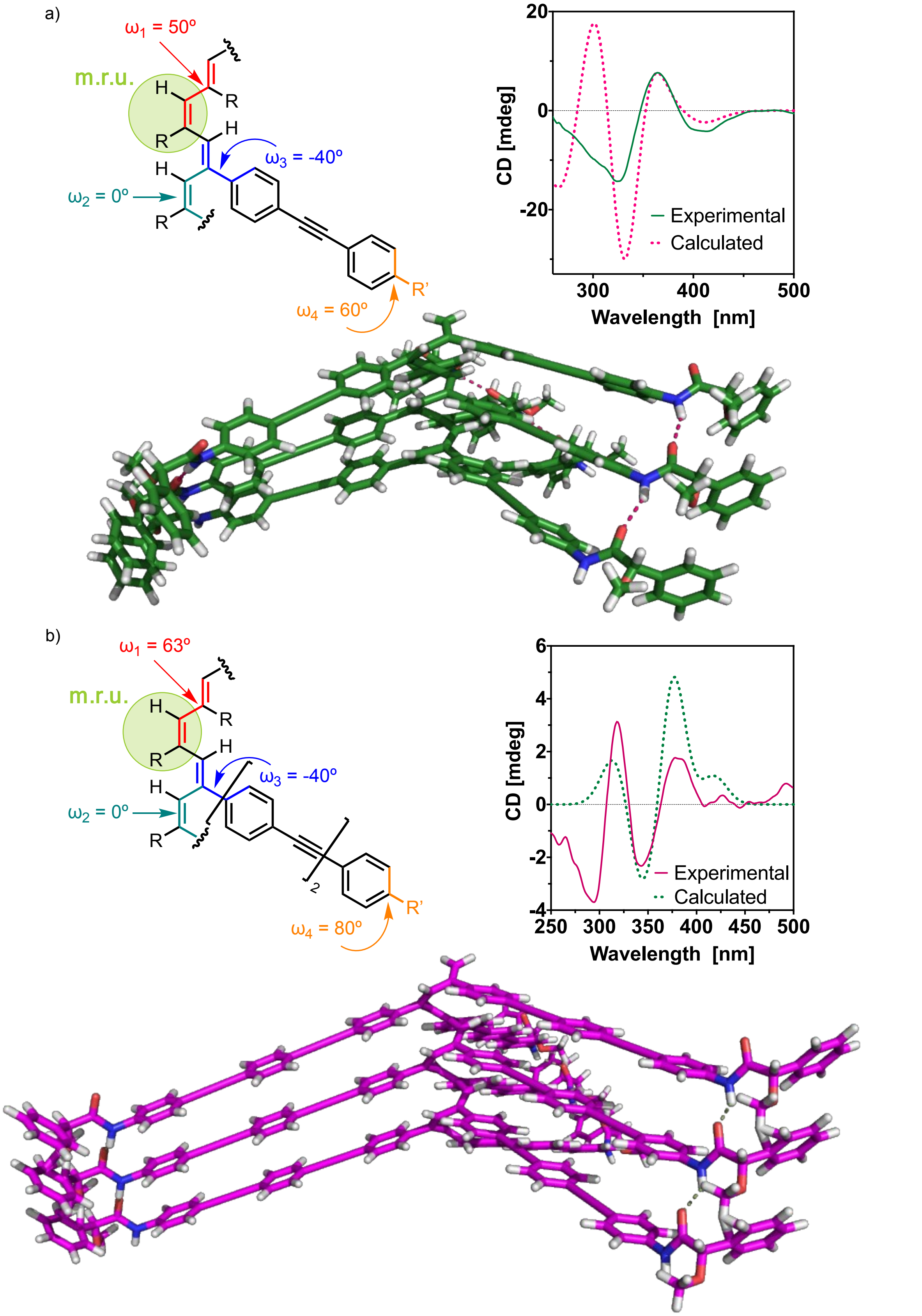

Figure S25. Dihedral angles used to build up the 3D model and the corresponding calculated CD (Full width at half-maximum (FWHM) equals 20 nm) for (a) poly-**2** and (b) poly-**3**.

13.2 Additional Computational Details

The methodology used to perform the theoretical calculations was selected according to the size of the polymers under investigation. Taking this into account, we selected the TD-DFT[S3] method and the CAM-B3LYP[S4] functional due to its a good performance in the evaluation of ECD spectra, as previously observed for theoretical calculations in PPAs.[48,49]

The 3-21G basis set was selected after performing a basis set selection study carried out on a PPA with n = 9, where this basis set proved to be a good choice to evaluate the spectra for larger polymers.

In order to compare the theoretical and experimental spectra, a correction factor was applied. The theoretical spectrum was adjusted to the experimental one by comparing the wavelength and intensity at the maximum/minimum corresponding to the first Cotton effect. We evaluated a correction factor for lambda as the difference between theoretical and experimental wavelengths (at first the maximum/minimum) and shifted the theoretical spectrum accordingly. Regarding the CD intensity, the theoretical values were rescaled in order to be able to compare them to the experimental ones.

14. Supporting References

S1. F. Freire, J. M. Seco, E. Quiñoá and R. Riguera, *Angew. Chem. Int. Ed.* 2011, **50**, 11692-11696.

S2. A. Rizzo, S. Coriani and K. Ruud *Computational Strategies for Spectroscopy. From Small Molecules to Nano Systems,* Barone, V. (Ed.) John Wiley & Sons, New Jersey 2012, **77**.

S3. E. Runge and E. K. U. Gross *Phys. Rev. Lett*. 1984, **52**, 997-1000.

S4. Y. Yanai, D. P. Tew and N. C. A. Handy *Chem. Phys. Lett.* 2005, **393**, 51-57.

S5. J. S. Binkley, J. A. Pople and W. J. Hehre *J. Am. Chem. Soc.* 1980, **102**, 939-947.

S6. F. Neese, *WIREs Comput Mol Sci*. 2012, **2**, 73-78.

S7. A. R. Allouche *J. Comput. Chem.* 2011, **32**, 174-182.
